# *Bacillus* seed coating mitigates early growth reduction in successive winter wheat without altering rhizosphere bacterial and archaeal communities

**DOI:** 10.1186/s12870-026-08128-2

**Published:** 2026-01-14

**Authors:** Nikolaos Kaloterakis, Andrea Braun-Kiewnick, Mehdi Rashtbari, Adriana Giongo, Doreen Babin, Priscilla M. Zamberlan, Bahar S. Razavi, Kornelia Smalla, Rüdiger Reichel, Nicolas Brüggemann

**Affiliations:** 1 Institute of Bio- and Geosciences, Agrosphere (IBG-3), Forschungszentrum Jülich GmbH, Jülich, 52428 Germany; 2 Department of Soil and Plant Microbiome, Institute of Phytopathology, Christian- Albrechts-University of Kiel, Kiel, 24118 Germany; 3 Institute for Epidemiology and Pathogen Diagnostics, Julius Kühn Institute (JKI) – Federal Research Centre for Cultivated Plants, Braunschweig, 38104 Germany

**Keywords:** Bacillus pumilus, Microbiome, Rhizosphere, Root growth, Rotational position, Rhizotron, Winter wheat, Zymography

## Abstract

The soil legacy of successively grown winter wheat (WW) often leads to lower plant growth and yield. In this study, we assessed the effect of *Bacillus pumilus* seed inoculation on the early growth of successively grown WW. We conducted an outdoor experiment using newly designed temperature-regulated rhizotrons. WW was grown in soil from two rotational positions, i.e., first WW after oilseed rape (W1) and second WW after oilseed rape (W2), until the end of tillering. We measured several plant and soil biochemical parameters. In addition, amplicons of the 16S rRNA gene were sequenced to account for bacterial and archaeal community shifts in the rhizosphere, and functional genes involved in the nitrogen cycle were quantified to estimate possible changes in N cycling due to *B. pumilus* inoculation. *B. pumilus* seed coating significantly compensated for the early growth reduction of W2, and this effect was primarily linked to changes in root plasticity with a higher root length density and a smaller specific root length in inoculated W2 compared with non-inoculated W2. There was a higher LAP activity in the rhizosphere of inoculated W2 plants than in the rhizosphere of non-inoculated W2 plants and this was followed by a reduction in soil NO_3_^−^, most probably due to an enhanced plant N uptake capacity. This was also shown in the increased potassium content of the inoculated W2 plants compared with their non-inoculated counterparts. *B. pumilus* seed coating did not influence the bacterial and archaeal alpha and beta diversity, but differential abundance analysis identified differences in the relative abundance of certain taxa between non-inoculated and inoculated W2. While *B. pumilus* seed coating significantly improved root growth and nutrient uptake in W2, this was not accompanied by a higher absolute abundance of bacterial or archaeal genes involved in N-cycling. Our study suggests that certain plant-beneficial microbes can reverse the negative plant-soil feedback in successive WW rotations and provides strong evidence of *B. pumilus* seed coating to promote WW productivity under such rotations.

**Supplementary Information:**

The online version contains supplementary material available at 10.1186/s12870-026-08128-2.

## Introduction

The potential of plant growth-promoting rhizobacteria (PGPR) in enhancing plant growth is receiving much attention, mainly due to their multifunctionality under different biotic and abiotic conditions [1]. This is depicted by the very high rates of annual growth of the biostimulants market, estimated at 13.58% in 2022 and projected to be worth approximately USD 5.6 billion by the end of 2025 [2]. The projected adverse climatic conditions of the following decades, with environmental concerns over the overuse of mineral fertilizers, create a unique opportunity to address decreasing crop yields by harnessing the potential of soil life on plant growth [3].

In the context of arable farming, the soil legacy following the growth of a particular plant species can affect the microbial community composition and the growth of the succeeding plant, which can result in negative, neutral, or positive plant-soil feedback (PSF) [4, 5]. Rhizodeposition is one of the most critical factors shaping the rhizobiome alongside soil abiotic properties [6, 7]. The released carbohydrates from root exudates and plant litter can stimulate the activity of PGPR in the rhizosphere and promote root growth and nutrient uptake, enhancing plant health and performance [8]. PGPR inoculation can improve soil quality by enhancing enzymatic activity, nitrogen (N) availability, and yield [9]. There is also evidence that certain PGPR, such as *Bacillus* spp. and *Trichoderma* spp., when used as inoculants, can alter the soil microbial community composition, increasing the relative abundance of microbes with antifungal or other plant-beneficial properties [10, 11]. One of the most common drawbacks of applying single-strain PGPR is that the beneficial properties observed in the lab or pot experiments often fail to translate into consistent trends under field conditions [12]. It is, therefore, crucial for the experimental conditions of potted experiments, which are the intermediate step between in vitro and field experiments, to resemble field climatic conditions.

Applying the PSF theory to crop rotations, the beneficial effect of oilseed rape as a preceding crop to winter wheat (WW) has been previously established [13–17]. Up to 40% of the global WW cultivation is grown successively, a practice that alters the rhizobiome and leads to significant yield losses, an effect further exacerbated by the build-up of soil-borne fungal pathogens such as *Gaeumannomyces tritic*i causing the take-all disease [18–22]. While, oilseed rape often reduces pathogen pressure (e.g., take-all) and restores yields, other legacy features such as the altered microbial community dynamics and available N pools may affect the growth of the subsequent WW [16, 23]. It is, therefore, important to research the beneficial preceding crops to WW and, at the same time, assess the potential of PGPR to restore productivity in the successively grown WW. Capitalizing on this knowledge, we can assume that, in conditions where the soil microbiome does not sufficiently support WW growth, the addition of a PGPR will promote plant health and productivity [1]. Previous experiments have shown that negative PSF are associated with major yield losses in successive WW rotations [24, 25]. These studies showed that soil mineral N is a key factor in explaining how WW rotations drive PSF and yield. Given the economic importance of WW, it is of utmost importance to research the potential of management practices to mitigate these yield losses. In a greenhouse experiment, seed inoculation with a promising *Bacillus* strain (*Bacillus pumilus* 45_39) significantly increased both root and shoot biomass of WW in both *Gaeumannomyces tritic*i-treated and untreated soil [26]. In addition to pathogen antagonism, it was also found capable of producing extracellular hydrolytic enzymes, namely proteases, cellulases and glucanases, making it a promising candidate strain for alleviating negative PSF in successive WW rotations. The effect of this strain on the microbial community, soil enzymatic activity and soil N dynamics remains unknown and could inform if and how *B. pumilus* inoculation can influence soil N availability and plant N uptake.

The research on PGPR functions and their effect on plant growth promotion is still in its infancy, with the vast majority of potential beneficial microbial candidates remaining unexplored. We studied the complex interactions between the rotational position of WW, *B. pumilus* seed coating and their interaction on soil mineral N, N-cycling, root adaptation and rhizobiome community structure, focusing on plant growth. Our study aimed to assess the effect of *B. pumilus* seed coating on the productivity of successive and non-successive WW rotations in an outdoor rhizotron experiment. We hypothesized that (i) *B. pumilus* seed coating would promote the root growth of the successional WW to a level similar to the non-successional WW, and this would be associated with a higher soil enzymatic activity, nutrient uptake, and plant performance, thus counteracting yield depression. (ii) A more efficient N supply and plant uptake in the *B. pumilus*-treated successional wheat (W2+) would be visible in the bacterial and archaeal community (higher abundance of bacteria involved in N-cycling) and in the absolute abundance of genes involved in N-cycling, enhancing N availability for WW uptake.

## Methods

### Experimental design

An outdoor rhizotron experiment was set up (June 6, 2023, to July 31, 2023) at Forschungszentrum Jülich, Germany, using newly designed rhizotrons (100 cm height, 35 cm width and 2.5 cm inner thickness) [27]. The rhizotrons were optimized for soil temperature regulation by circulating 5 °C water through the upper polyvinyl chloride plates connected to a cooling unit. The rhizotrons were connected to the cooling unit with polyurethane tubing coated with a rubber-insulating hose. To create a realistic soil temperature gradient along the rhizotron, the water was first directed into the lower side of the rhizotron and flowed upwards to the rhizotron/soil surface. Preliminary trials showed that the temperature changes during the upward movement of the cooling water through the rhizotron material created a realistic temperature gradient in the soil profile. Passive insulation was provided by fixing an insulating 4 cm layer, consisting of a 1 cm thick rubber and a 3 cm thick Styrofoam™ layer, on both plates of the rhizotrons (Fig. A1). Throughout the experiment, one rhizotron was dedicated to measuring soil temperature at three soil depths, i.e., 10 cm, 40 cm, and 80 cm. Ambient weather conditions and soil temperature gradient along the soil profile of the rhizotron are shown in Fig. A2.

### Soil origin and rotational positions of wheat

To simulate the first WW after oilseed rape (W1) and the second WW after oilseed rape (W2), we used soil after one season of oilseed rape cultivation and after one season of WW, collected from the experimental farm Hohenschulen, Faculty of Agricultural and Nutritional Sciences, Christian-Albrechts-University of Kiel, 54°19′05″N, 9°58′38″E, Germany. The crop rotation trial was established in 1989. It included the following factors: (a) rotational position (oilseed rape, first wheat after oilseed rape, second and third wheat grown successively), (b) WW varieties (4 genotypes), and (c) N fertilization levels (4 levels). Composite soil samples for the experiment were taken from the topsoil (0–30 cm) and subsoil (30–50 cm) of field plots after oilseed rape and after one season of WW cultivation (*n* = 4 replications) containing the WW cultivar “Nordkap” (*Triticum aestivum* L., seed source: SAATEN-UNION GmbH, Isernhagen, Germany) and optimal N-fertilization (240 kg N ha^− 1^). After the different preceding crops were harvested, the plant residues remained on the field. The residues of the preceding crops were not removed from the soil before sampling, and the field was ploughed after the sampling. The soil is a Cambic Luvisol of sandy loam texture (44% sand, 35% silt, and 21% clay) with no carbonates [28, 29]. The soil properties of the specific plots from which the soil was taken have been previously described in Kaloterakis et al. [17].

### Soil conditioning, seed inoculation and plant growth conditions

The fresh soil was sieved to 2 mm and repacked in the rhizotrons to reach a bulk density of 1.33 g cm^− 3^ and 1.45 g cm^− 3^ for 0–30 cm and 30–100 cm, respectively. Deionized water was added to adjust soil moisture from 50% to 70% water-holding capacity (215 g H_2_O soil kg^− 1^) at the onset of the experiment. Thereafter, the plants were kept rain-fed throughout the experiment. All rhizotrons were kept inclined at 45^ᵒ^ to facilitate root growth along the lower side of the rhizotrons. WW seeds (cultivar “Nordkap”) were inoculated with either *B. pumilus* 45_39 (DSM117807, DSMZ, Braunschweig, Germany) or buffer solution (control) as described in Braun-Kiewnick et al. [26]. Briefly, seeds were pre-germinated at room temperature for two days in the dark and then immersed in cell suspensions of *B. pumilus* vegetative cells from fresh overnight cultures. Bacterial cultures were grown in 50 mL R2A broth containing 100 µg mL^− 1^ rifampicin in 300 mL Erlenmeyer flasks at 150 rpm and harvested by centrifugation (5000 *g*, 15 min at room temperature) using 50 mL conical tubes and washing three times in 10 mM MgCl_2_ buffer. The cell pellet was resuspended in 5 mL 10 mM MgCl_2,_ and 5 mL of 2% (v/v) sterile methyl cellulose was added as a coating agent to both inoculated and non-inoculated seeds. Approximately twenty-five pre-germinated seeds were submerged and soaked in the 10 mL cell suspension for one hour. The tubes were gently shaken throughout soaking to ensure even contact between each seed and the cell suspension. One seed was added to 1 mL 10 mM phosphate buffered saline (PBS) per replication (*n* = 3 replications) after coating and the solution was then serially diluted to 10^− 7^. Colony-forming unit (CFU) counts conducted directly after seed treatment resulted in 1.32 × 10^9^ CFU (Log 9.12) per seed. Three WW seeds were sown in each rhizotron and thinned to one seedling per rhizotron 3 days after sowing. The plants were not fertilized for the duration of the experiment. The plants were harvested at the end of the tillering stage (BBCH 29, BBCH from the Biologische Bundesanstalt, Bundessortenamt und CHemical Industry decimal code system).

### Above and belowground plant growth analyses

The aerial plant parts were split at harvest (BBCH 29) into pseudostems (hereafter called stems) and leaves. The lower sides of the rhizotrons were then removed, and the soil profile was divided into three layers (0–30 cm, 30–60 cm, and 60–100 cm). We pooled and mixed subsamples to form a composite sample and then split it into several parts to ensure enough soil was collected for every planned analysis. Rhizosphere soil was collected at plant harvest as described in Braun-Kiewnick et al. [26]. Briefly, loosely adhered soil was vigorously shaken off. The roots with the rhizosphere soil still attached were placed on a sterile surface, and the rhizosphere was gently brushed off the root surface with a disposable toothbrush. Soil samples were stored at −25 °C before analysis of mineral N, dissolved organic carbon (DOC), and total extractable nitrogen (TN) and at 4 °C before enzymatic analysis and soil DNA isolation.

After collecting the rhizosphere soil, the roots were washed and stored in 30% ethanol. They were scanned at 600 dpi (Epson Perfection V800 Photo, Epson, Japan) and analyzed with WinRhizo^®^ software (Regent Instruments Inc., Quebec, Canada). Next, they were split into six diameter (R_dia_) classes, i.e., ≤ 0.1 mm, 0.1–0.2 mm, 0.2–0.3 mm, 0.3–0.4 mm, 0.4–0.5 mm, ≥ 0.5 mm. All plant materials were oven-dried at 60 °C to constant weight (maximum three days) to record their dry weight. Ball-milled (MM 400, Retsch, Germany) above and belowground plant samples, as well as soil samples, were weighed into tin capsules (HEKAtech, Wegberg, Germany) for the determination of C and N content using an elemental analyzer coupled to an isotope-ratio mass spectrometer (EA-IRMS, Flash EA 2000, coupled to Delta V Plus; Thermo Fisher Scientific Inc., Waltham, MA, USA). Microwave digestions of ball-milled plant material in closed vessels were performed and measured for phosphorus (P), potassium (K), and iron (Fe) content by inductively coupled plasma optical emission spectroscopy (ICP-OES; iCAP 7600; Thermo Fisher Scientific Inc., Waltham, MA, USA; adjusted from Hansen et al. [30]).

### Soil sample processing

For soil nutrients quantification, soil samples were extracted using 0.01 M CaCl_2_ (soil-to-solution ratio of 1:4 w:v), vortexed, shaken horizontally for 2 h at 200 rpm, centrifuged for 15 min at 690 × *g*, and filtered through 0.45 μm PP-membrane filters (Ø 25 mm; DISSOLUTION ACCESSORIES, ProSense B.V., Munich, Germany). Soil solution was stored overnight at 4 °C before DOC and TN analysis. Ammonium (NH_4_^+^) was measured by continuous-flow analysis (Flowsys, Alliance Instruments GmbH, Freilassing, Germany). Nitrate (NO_3_^−^) and sulfate (SO_4_^2−^) were measured by ion chromatography (Metrohm 850 Professional IC Anion – MCS, Metrohm AG, Herisau, Switzerland). Mg was measured by ICP-OES (iCAP 6500; Thermo Fisher Scientific Inc., Waltham, MA, USA). The pH was measured in the same solution using a glass pH electrode (SenTix^®^ 940, WTW, Xylem Analytics, Weilheim, Germany). DOC and TN were quantified with a total organic C (TOC) analyzer (TOC-V + ASI-V + TNM, Shimadzu, Japan). Soil enzymatic analysis and zymography were performed as in Kaloterakis et al. [17].

### Soil DNA extraction

Total DNA from 48 rhizosphere samples was extracted using 0.5 g of soil with the FastDNA SpinKit for Soil (MP Biomedicals, Eschwege, Germany) and the FastPrep-24 bead beater according to the manufacturer’s instructions and following established protocols [22]. The concentration and integrity of the total DNA were checked by Nanodrop (Thermo Fisher, Waltham, USA) and on 0.8% agarose gels, respectively.

###  16S rRNA gene amplicon sequencing and sequence analyses

The amplicon libraries were generated using primers Uni341F (5’ CCTAYGGGRBGCASCAG 3’) and Uni806R (5’ GGACTACNNGGGTATCTAAT 3′; Sundberg et al. 2013) with Illumina adaptors (Illumina, San Diego, USA) and sequenced at Novogene (UK) using Illumina MiSeq v2 (2 × 250 bp) chemistry according to the manufacturer’s instructions.

The downstream analysis was conducted in R (v4.2.1 [31]). Sequence analysis utilized the DADA2 v1.16.0 pipeline [32], with the following modifications: trimLeft = c(17,20) and no truncation. In total, 3,489,733 sequences were obtained from 96 samples. Taxonomic classification of amplicon sequence variants (ASVs) employed the SILVA database v.138 [33] and the phyloseq package [34]. The function “subset_taxa” from the phyloseq package was used to remove any residual ASV identified as chloroplasts, mitochondria, or eukaryotes and ASVs unassigned at the phylum level. A taxonomic level called “Annotation” was manually included in the table, then processed using a “tax.clean” function from the phyloseq package that replaces NA with the latest taxonomy found for an ASV. The dataset was processed to yield an even level of sequencing by rarefaction to the fewest sequences found in all samples [35]. It resulted in a minimum of 36,361 sequences per sample in the dataset. Alpha diversity indices were computed using phyloseq and microbiome packages [36], with Kruskal–Wallis tests and post-hoc Wilcoxon–Mann–Whitney tests conducted for significant changes. Beta diversity analysis utilized square-root transformed ASV count data, and the Bray-Curtis dissimilarity index was computed to generate a distance matrix between the samples using the ‘vegan’ package [37]. Differences in beta diversity centroids were assessed using permutational multivariate analysis of variance (adonis), PERMANOVA tests, and ANOSIM. Differential abundance (DA) analysis employed the DESeq2 package [38] within phyloseq, at the annotation level, for the ones considered significant at adjusted *p*-value < 0.05 after Benjamini-Hochberg correction.

### Quantitative qPCR (bacterial and archaeal genes)

Quantitative PCR (qPCR) was done as described previously [24]. Briefly, qPCRs measured the absolute abundance of bacterial 16S rRNA gene copies and N cycle-related genes, including the ammonia monooxygenase (AMO) alpha subunit (*amoA*), NO_2_^−^ reductase (*nirS*), nitrous oxide reductase (*nosZ*), and N_2_-fixing nitrogenase (*nifH*) genes. To quantify absolute bacterial abundance, the BACT1369F, PROK1492R, and TM1389F primers, labeled with 5’-FAM and 3’-TAMRA, were used in a specific TaqMan assay, following the protocol described by Suzuki et al. [39]. To determine the absolute abundance of the bacterial *amoA* gene (AOB) the primers *amoA*1 F and *amoA*2 R developed by Rotthauwe et al. [40] were employed as described in Meyer et al. [41]. For quantifying the *nirS* gene, the primers cd3aF and R3cd [42] and for the *nifH* gene, the forward primer FPGH19 [43] was used along with the reverse primer PolR [44]. Finally, for the *nosZ* gene quantification, the *nosZ*−2 F and *nosZ*−2R primers were used [45]. All reactions were conducted in a total volume of 20 µL containing 10 µL of 2 x Luna Universal qPCR Master Mix (New England Biolabs, Ipswich, USA), 0.1 mg mL^− 1^ BSA, 200 nM (*amo*A) or 400 nM (*nir*S, *nif*H, *nos*Z) of each primer and 5 µL of template DNA (1:5 diluted) per reaction. PCR cycling conditions were 1 min at 95 °C, followed by 40 cycles of 15 s at 95 °C and 30 s at 60 °C. The specificity of the amplification products employing SYBR green chemistry (New England Biolabs, Ipswich, USA) was confirmed through melting curve analyses after completion of amplification cycles (60–95 °C (𝜟 0.5 °C every 5 s)). The quantification of all target gene copies in the samples was determined by comparing them to standard curves of each gene separately. Cloned and purified target gene copies, and all measurements were based on 1 g of soil (gene copies per g of soil). Standard curves were generated by serial dilutions of target genes (ranging from 10^−2^ to 10^−7^). Reference DNAs for bacterial *nirS*, *nifH*, nosZ, and *amoA* genes were used based on purified gene fragments inserted into either the pEASY-T1 (*nir*S, *nif*H, *nos*Z) or pCR2.1 (*amo*A) cloning vectors and transformed into *E. coli*. All measurements were run in duplicates (= technical replicates) on a CFX96 Real-Time System (Bio-Rad, Laboratories GmbH, Munich, Germany). Precautions were taken to ensure that the data from each duplicate fell within 0.5 threshold cycle (Ct), and clear outliers (*>* 2 standard deviations) were removed before calculating the average Ct of each treatment, with each treatment having three biological replicates, resulting in six data points per measurement. Melting curves and non-template controls were used to assess run reliability. There was no detectable amplification arising from non-template controls in any of the assays. The amplification efficiencies of all qPCR assays ranged from 91% to 98% and was calculated from the formula: Efficiency (%) = $\:{\left(\right(10}^{\frac{-1}{slope}})-1)\times\:100$ by the qPCR machine.

For quantification of total Archaea and *amo*A (AOA) gene copies of Archaea, Archaea-specific qPCRs were conducted. To quantify the absolute abundance of Archaea, the ARC787F_YU, ARC1059R_YU, and ARC915F_YU probes (with 5’-FAM and 3’-TAMRA labels) were used in a TaqMan assay, following the protocol described by Yu et al. (2005). For quantification of the archaeal ammonia monooxygenase alpha subunit (*amoA*), the forward primer amo19F [46] and reverse primer CrenamoA616r48x [47] were used according to the protocol described by Meyer et al. [41]. PCR reactions were conducted in a total volume of 20 µL containing 10 µL of 2 x Luna Universal qPCR Master Mix (New England Biolabs, Ipswich, USA), 0.1 mg mL^− 1^ BSA, 600 nM of each primer and 5 µL of template DNA (1:5 diluted, ca. 10–20 ng µL^− 1^) per reaction. PCR cycling conditions were 10 min at 94 °C, followed by 40 cycles of 45 s at 94 °C, 45 s at 60 °C, and 45 s at 72 °C. The specificity of amplification products employing SYBR green chemistry was confirmed through melting curve analyses after completion of amplification cycles (72–95 °C (𝜟 0.5 °C every 5 s)). The quantification of target gene copies was conducted as described above and reference DNA for total Archaea was based on 10-fold serial dilutions of purified PCR product 16S rRNA gene from *Methanobacterium oryzae* (ca 1300 bp, Thünen Institute, AG Tebbe, cloned into pGEM-T, transformed in *E*. *coli*). Reference DNA for the archaeal *amo*A gene (AOA) was based on 10-fold serial dilutions of purified archaeal *amo*A gene fosmid clone 54d9 (656 bp) from *Crenarchaeota* (Helmholtz Zentrum München, AG Schloter/V. Radl) cloned into pCR2.1 and transformed into *E. coli*. Calculations of total Archaea and AOA gene copy numbers were done as described above.

### Statistical analysis for soil and plant parameters

The rotational position of WW (W1 and W2), PGPR inoculation status (‘+’ and ‘-‘), soil depth (0–30 cm, 30–60 cm, and 60–100 cm), plant part (root, stem, leaf) and their interactions were considered fixed factors and the replicates were considered random factors in the statistical analysis. To account for normality violations and unequal variances of some variables, we identified the highest fitting distribution for each response variable by comparing gamma, Gaussian and log-normal distributions. We fitted three models: a generalized linear mixed model (GLMM) where the gamma distribution was the highest fit, a linear mixed-effects model (LMM) for variables following a Gaussian distribution and an LMM using log-transformed data. The respective model is mentioned in the respective graph and table. Model selection was based on the Akaike Information Criterion and the Bayesian Information Criterion. Type III ANOVA (Wald chi-square tests) was conducted to assess the significance of the fixed factors and post-hoc tests with Benjamini-Hochberg p-value adjustment of estimated marginal means were performed using the ‘emmeans’ package [48]. Pairwise tests were computed across all soil depths and/or plant parts and within each depth and or plant part. All analyses were performed in R (v4.2.1 [31]).,. Graphics were generated using ggplot2 [49] unless otherwise specified. To assess how WW rotation, PGPR inoculation and their interaction influence soil microbial communities, soil parameters and plant growth traits, we performed a redundancy analysis (RDA) using the ‘vegan’ package. The following bacterial and archaeal taxa (36 taxa) that were previously shown to be significantly recruited by W1 and W2 [24], taxa that were screened for plant-beneficial traits [26] in the same rotations, and important N-cycling taxa were included in the analysis: *Nitrosomonadaceae*, *Nitrososphaeraceae*, *Nitrospiraceae*, *Rhodanobacteraceae*, Acidobacteriales, *Bacteroidaceae*, *Oxalobacteraceae*, *Rhizobiaceae*, *Rhodobacteraceae*, *Xanthobacteraceae*, *Xanthomonadaceae*, *Arthrobacter*, *Microbacterium*, ANPR, Candidatus_Udaeobacter, *Caulobacter*, *Flavobacterium*, *Gaiella*, *Gemmatimonas*, KD3_93, *Lysobacter*, MB_A2_108, *Methyloligellaceae*, *Pseudomonas*, *Pedobacter*, *Phenylobacterium*, *Shinella*, Sphingomonas, *Stenotrophomonas*, *Streptomyces*, *Tumebacillus*, *Variovorax*, *Bacillus*, *Devosia*, *Neisseria* and *Paenibacillus*. The relative abundance of microbial (bacterial and archaeal) data was Hellinger-transformed and the remaining variables were standardized (Z-score) before the analysis. Model significance was evaluated with permutation tests using 999 permutations. The highest influencing variables for RD1 and RD2 were identified based on their loading on the respective RD.

## Results

### Plant growth and nutrient uptake

**Fig. 1:**
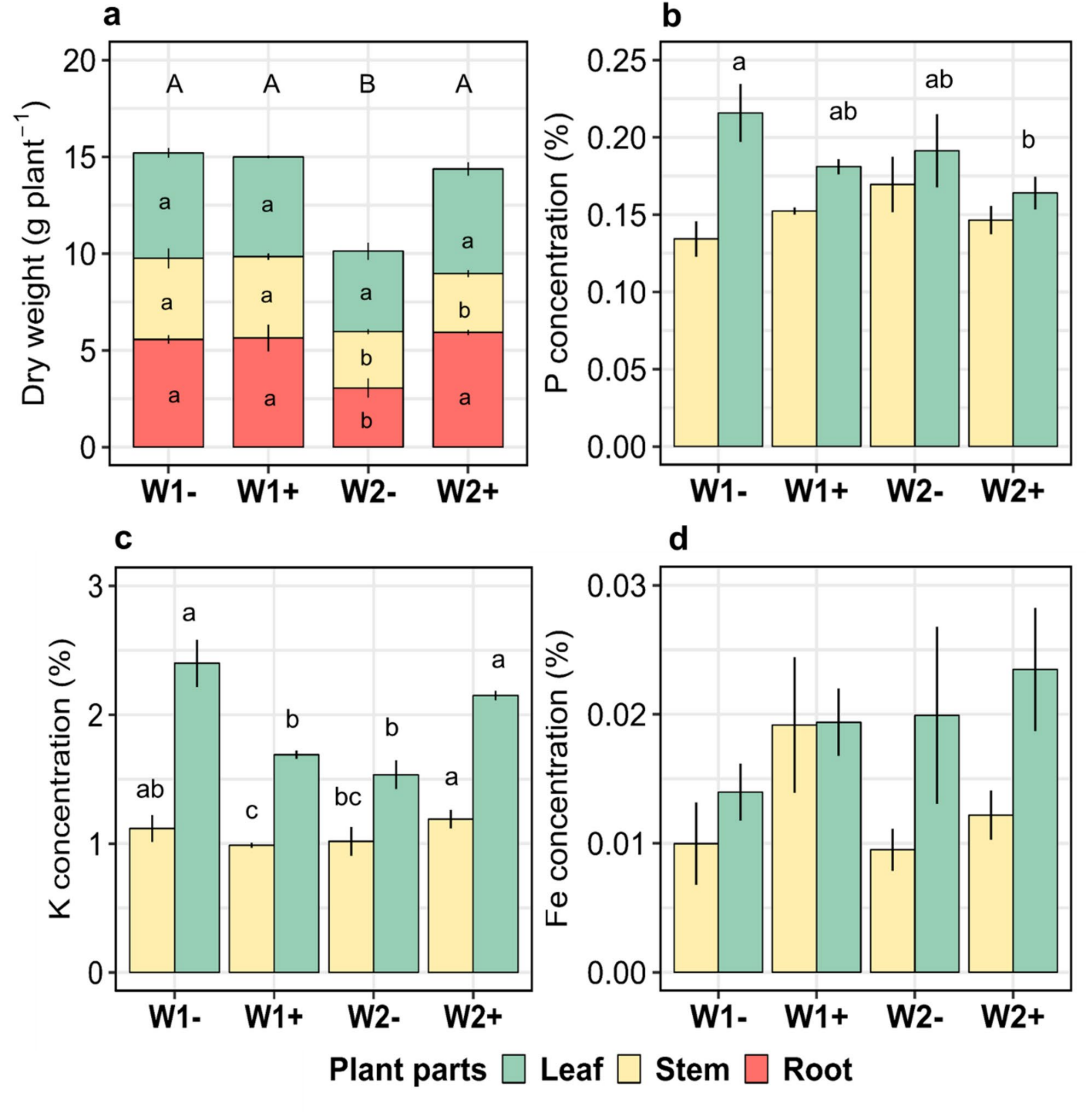
Plant dry weight (**a**), P concentration (**b**), K concentration (**c**), and Fe concentration (**d**) of roots, stems and leaves of winter wheat at the end of tillering (BBCH 29). First wheat after oilseed rape without (W1-) and with (W1+) *Bacillus pumilus* and second wheat after oilseed rape without (W2-) and with (W2+) *Bacillus pumilus*. Different uppercase letters indicate significant differences between the rotational positions and PGPR treatments across all plant parts. Plant dry weight was analyzed using LMM. For P, K and Fe we used GLMM with gamma distribution. Within each plant part, different lowercase letters indicate significant differences between the rotational positions and PGPR treatments at *p* ≤ 0.05 using the Benjamini-Hochberg adjustment for multiple comparisons. The absence of letters indicates non-significant differences

Rotational position strongly influenced root biomass accumulation and plant nutrient content at tillering (Table A1). There was a significant interaction between WW’s rotational position, PGPR treatment and the plant part. W1- produced a 52.9% higher total plant biomass than W2- (Fig. 1a). *B. pumilus* seed coating increased total plant biomass in W2 + but not in W1+, with respect to their non-inoculated counterparts. For W2+, the bacterial inoculation resulted in the doubling of root biomass (Fig. 1a). Plant tissue analysis showed that the most significant differences were shown for N% and K% in the aboveground plant parts (Table A1). There was a decreasing P% trend in the leaves of *B. pumilus* inoculated plants, which was, however, not significant (Fig. 1b). A 27.5% decrease in K% of the aboveground biomass in W2- compared with W1- was shown (Fig. 1c), which was evident in both leaves and stems. *B. pumilus* seed coating led to a higher K uptake in W2 + by increasing the K% by 30.9% in the shoot parts. Also, Fe% content in leaves and stems showed an increasing trend in *B. pumilus* inoculated rotations, which was not significant due to high variation (Fig. 1d). Pairwise comparisons revealed a higher plant N% in W1- compared with W2- with no obvious differences between their inoculated counterparts (Fig. A3b). This was partially reflected in the higher plant C:N ratio of W2- compared with W1- (Fig. A3c). *B. pumilus* seed coating also significantly decreased plant C:N ratio of W2 + compared with W2-.

**Fig. 2:**
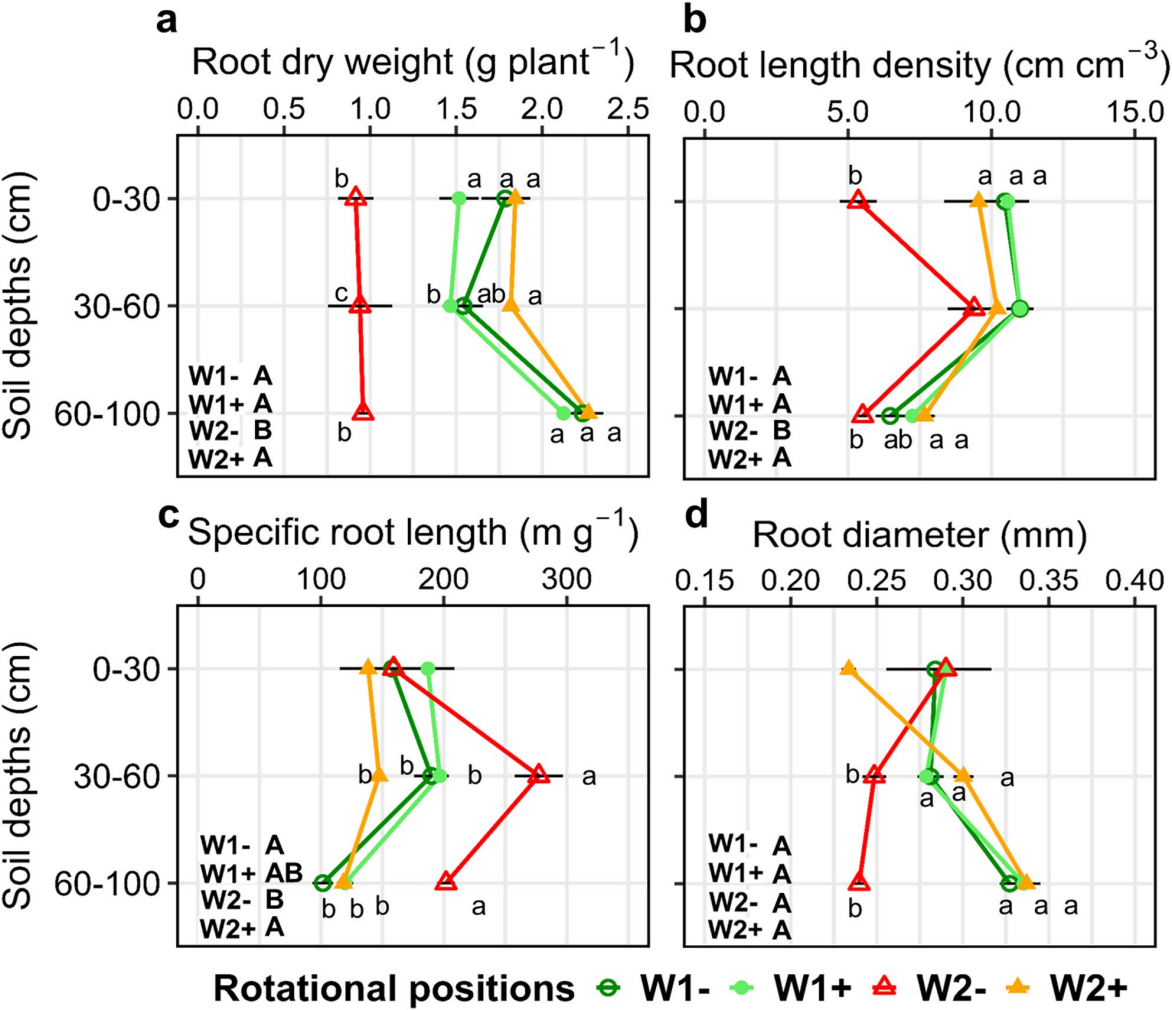
Root dry weight (**a**), root length density (**b**), specific root length (**c**) and average root diameter (**d**) of roots, root dry weight (**c**), and root length density (**d**) of two rotational positions of winter wheat at the end of tillering (BBCH 29) at soil depths 0–30 cm, 30–60 cm, and 60–100 cm. First wheat after oilseed rape without (W1-) and with (W1+) *Bacillus pumilus* and second wheat after oilseed rape without (W2-) and with (W2+) *Bacillus pumilus*. Different uppercase letters indicate significant differences between the rotational positions and PGPR treatments across all soil depths. SRL was analyzed using LMM. For RDW, R_dia_ and RLD, we used LMM with log-normal distribution. Within each soil depth, different lowercase letters indicate significant differences between the rotational positions and PGPR treatments at *p* ≤ 0.05 using the Benjamini-Hochberg adjustment for multiple comparisons. The absence of letters indicates non-significant differences

Root growth followed a similar pattern as plant aboveground dry weight, with W2- having significantly less root biomass compared with W1- and *B. pumilus* seed coating compensating this reduction (Table A2, Fig. 2a). W2- plants had on average 48.7%, 49.4% and 43.9% lower root length density (RLD) compared with W1-, W1 + and W2 + respectively which was evident across all three soil depths (Fig. 2b). Specific root length (SRL) was also significantly affected by WW’s rotational position (Table A2) with successively grown W2- having higher SRL than W1-, W1 + and W2 + in the 30–60 cm and 60–100 cm (Fig. 2c). Across all depths, W2- plants produced the thinnest roots with an average of 0.259 mm thick roots compared with 0.297 mm, 0.304 mm and 0.290 mm of W1-, W1 + and W2+, respectively (Fig. 2d). Successive WW inoculated with *B. pumilus* had thinner roots in the topsoil and thicker roots in both subsoil depths compared with non-inoculated successive WW. The proportion of root length from six R_dia_ classes supported this finding, with a higher proportion of thicker roots in W2 + compared with W2- (Table A3).

### Soil enzymatic activity and nutrient content

**Fig. 3:**
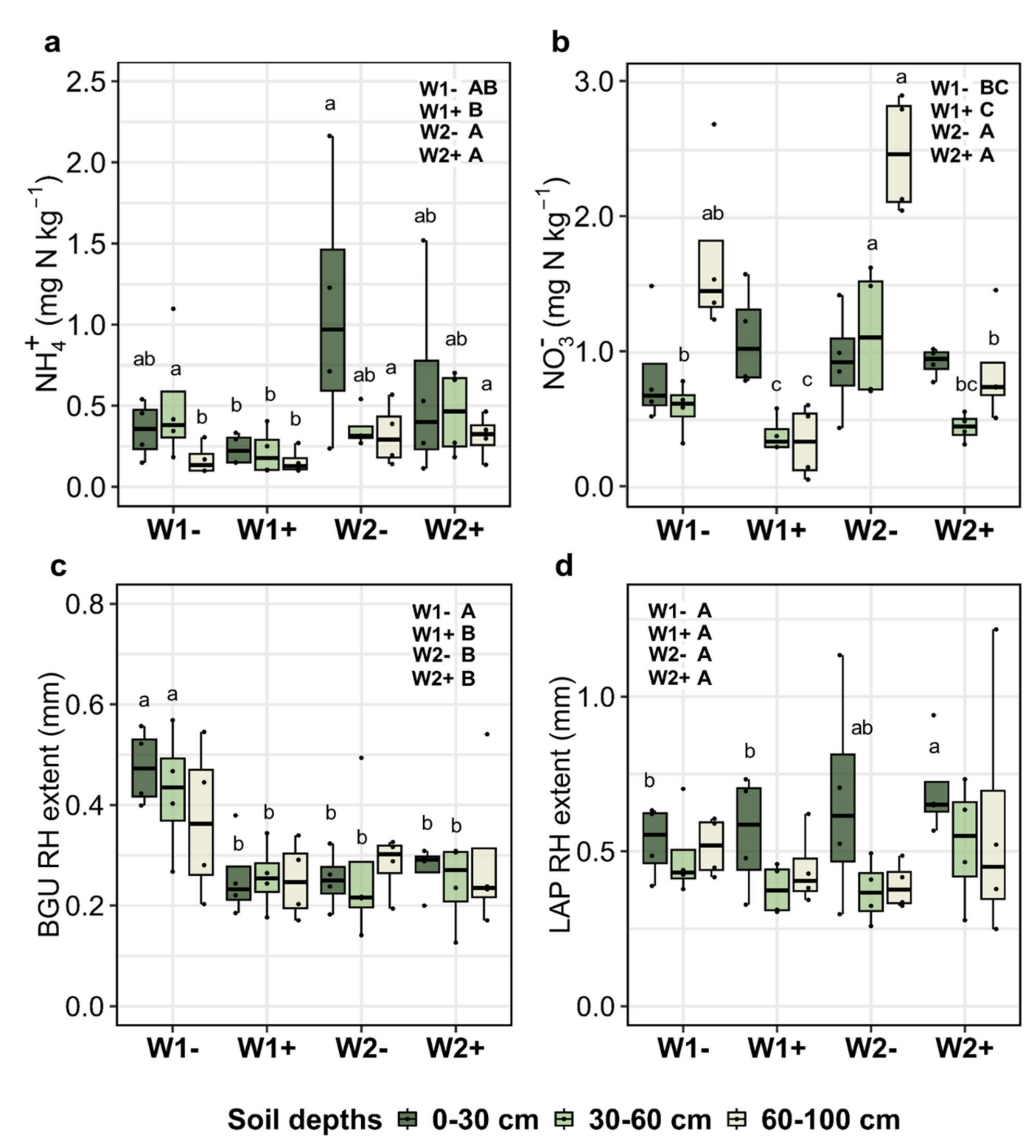
Effect of the rotational positions on soil NH_4_^+^-N (**a**), soil NO_3_^−^-N (**b**), rhizosphere (RH) extent of *β*-glucosidase (BGU; **c**) and RH extent of leucine aminopeptidase (LAP; **d**) of the following winter wheat at the end of tillering (BBCH 29) at soil depths 0–30 cm, 30–60 cm and 60–100 cm. First wheat after oilseed rape without (W1-) and with (W1+) *Bacillus pumilus* and second wheat after oilseed rape without (W2-) and with (W2+) *Bacillus pumilus*. Different uppercase letters indicate significant differences between the rotational positions and PGPR treatments across all soil depths. SRL was analyzed using LMM. Soil NH_4_^+^-N and NO_3_^−^-N, BGU extent and LAP extent were analyzed using GLMM with gamma distribution. Within each soil depth, different lowercase letters indicate significant differences between the rotational positions and PGPR treatments at *p* ≤ 0.05 using the Benjamini-Hochberg adjustment for multiple comparisons. The absence of letters indicates non-significant differences

Rotational position markedly impacted soil mineral N (especially NH_4_^+^), but not DOC (Table A4). We measured 71.6% higher soil NH_4_^+^ in inoculated and non-inoculated W2 plants compared with W1 which was mainly due to the 0–30 cm and 60–100 cm soil layers (Fig. 3a). *B. pumilus* seed coating reduced the soil NH_4_^+^ in the subsoil of W1 + compared with W1- but not in W2 + compared with W2- (Fig. 3a). There was a 44.5% higher soil NO_3_^−^ in W2- compared with W1- at tillering (Fig. 3b). We measured less soil NO_3_^−^ in the inoculated W1 + and W2 + than in their non-inoculated counterparts, which was especially evident in the 60–100 cm soil layers (−80.3% and − 65% reduction compared with W1- and W2-, respectively). A higher soil content of NH_4_^+^ was measured in the topsoil compared with NO_3_^−^ which was measured at higher concentrations in the 60–100 cm soil layer (Table A4; Fig. 3).

Concerning soil enzymatic activity, soil depth affected the kinetic parameters of BGU and LAP as well as their temporal variation, visualized with zymography (Table A5). The BGU rhizosphere extent of W1- was on average 0.42 mm compared with 0.27 mm rhizosphere extent of W2- (Fig. 3c). *B. pumilus* seed coating reduced BGU rhizosphere extent in W1 + compared with W1- but did not induce changes in W2. A higher rhizosphere extent for LAP (0.70 mm) across all soil depths was observed in W2 + compared with W2- (0.67 mm, albeit not significant), W1- (0.53 mm) and W1+ (0.56 mm; Fig. 3d). There were no differences in BGU V_max_ among the rotations and PGPR treatment overall, however, W2- had significantly higher BGU V_max_ compared with all other rotations with and without *B. pumilus* seed coating (Fig. A4a). LAP V_max_ was also significantly higher in W2 + compared with all other treatments, with no significant pairwise differences in each soil depth (Fig. A4b). BGU K_m_ values were significantly affected by the interaction of rotational position and *B. pumilus* seed coating (Table A5), with a lower BGU K_m_ for W2 + in the 60–100 cm compared with all other treatments, which was not the case for LAP K_m_.

### Microbial diversity and community structure

We did not observe significant differences in alpha diversity (Shannon, Chao1 and Pielou indices) between the microbial communities of W1 and W2 at tillering (Fig. A5). Depth reduced the microbial species diversity and richness, but not evenness, while the effect of rotational position of WW and/or PGPR treatment was insignificant (Fig. A5). PERMANOVA analysis revealed that depth and rotation had a significant influence on the microbial communities with depth explaining 15.4% of the variation between communities and rotation approximately 5.4%, with no effect of the *B. pumilus* seed coating, which explained only 1.6% of the variation. This was reflected in the closer clustering of the microbial communities by depth in W1 than in W2, with no clustering observed due to *B. pumilus* seed coating (Fig. A6).

**Fig. 4:**
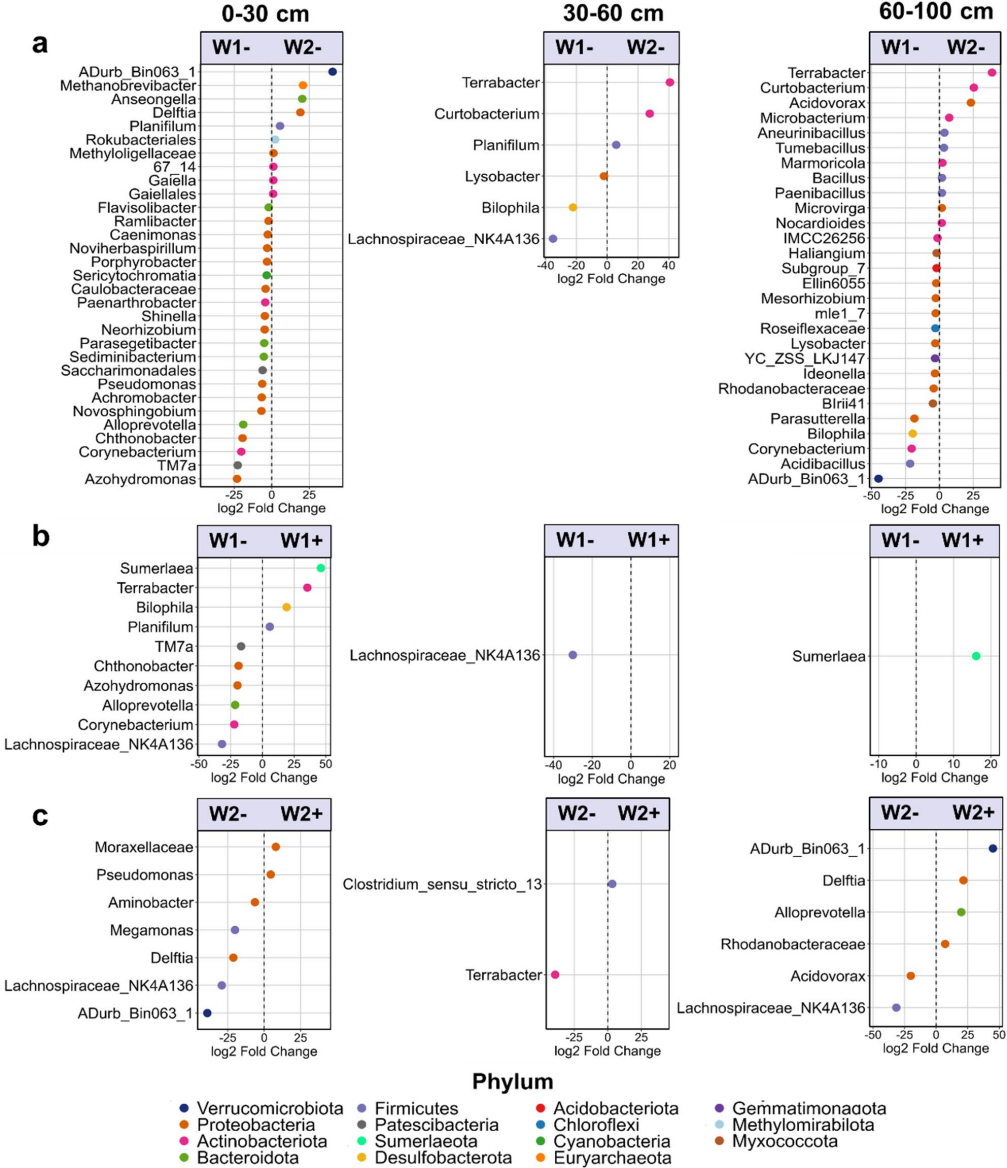
Differentially abundant (DA) taxa between (**a**) first (W1-) and second (W2-) wheat after oilseed rape without *Bacillus pumilus*, (**b**) W1- and first (W1+) wheat after oilseed rape with *Bacillus pumilus* and (**c**) W2- and second (W2+) wheat after oilseed rape with *Bacillus pumilus* at the end of tillering (BBCH 29), separated by soil depth (0–30 cm; 30–60 cm and 60–100 cm). Taxa with significant p values (threshold ≤ 0.05) were considered significantly DA. P values were adjusted using the Benjamini-Hochberg correction for multiple comparisons. For each taxon, the corresponding phylum is represented by a unique color

When we compared the influence of WW rotational position and PGPR treatment on the microbial communities across the three soil layers, we found that more taxa were differentially abundant at 0–30 cm and 60–100 cm compared with 30–60 cm (Fig. 4). When comparing W1- and W2- across the three soil depths, we found that twenty-one taxa were more abundant in W1- and ten taxa were more abundant in W2- in the topsoil (Fig. 4a). Among them, we found a higher abundance of *Noviherbaspirillum*, *Caulobacteraceae*, *Porphyrobacter*, Saccharimonadales, *Pseudomonas*, *Paenarthrobacter*, *Achromobacter*, *Corynebacterium* and TM7a in W1- and a higher abundance of ADurb.Bin063-1, *Delftia* and *Planifilum* in W2- at 0–30 cm. At 30–60 cm, we found that *Lachnospiraceae* NK4A136, *Lysobacter* and *Bilophila* were more abundant in W1-, while *Terrabacter*, *Curtobacterium* and *Planifilum* were more abundant in W2-. Seventeen taxa were more abundant in W1-, and eleven more abundant in W2-, at 60–100 cm. Among them, *Rhodanobacteraceae*, *Bilophila*, *Corynebacterium* and ADurb. Bin063-1 were more abundant in W1- and *Terrabacter*, *Curtobacterium*, *Acidovorax*, *Bacillus*, *Paenibacillus* were more abundant in W2-. When we assessed the effect of *B. pumilus* seed coating on W1, we found a higher abundance of *Sumerlaea* in W1 + compared with W1- at both 0–30 cm and 60–100 cm (Fig. 4b). Most of the differences were observed at 0–30 cm, with six taxa more abundant in W1- and four taxa more abundant in W1+. When we compared W2- and W2 + we observed five taxa more abundant in W2- and two taxa (*Moraxellaceae* and *Pseudomonas*) more abundant in W2 + at 0–30 cm (Fig. 4c). Among others, we found that *Acidovorax* was more abundant in W2- and *Rhodanobacteraceae* were more abundant in W2 + at 60–100 cm.

### Redundancy analysis of plant growth traits, soil properties and rhizobiome community shape

**Fig. 5:**
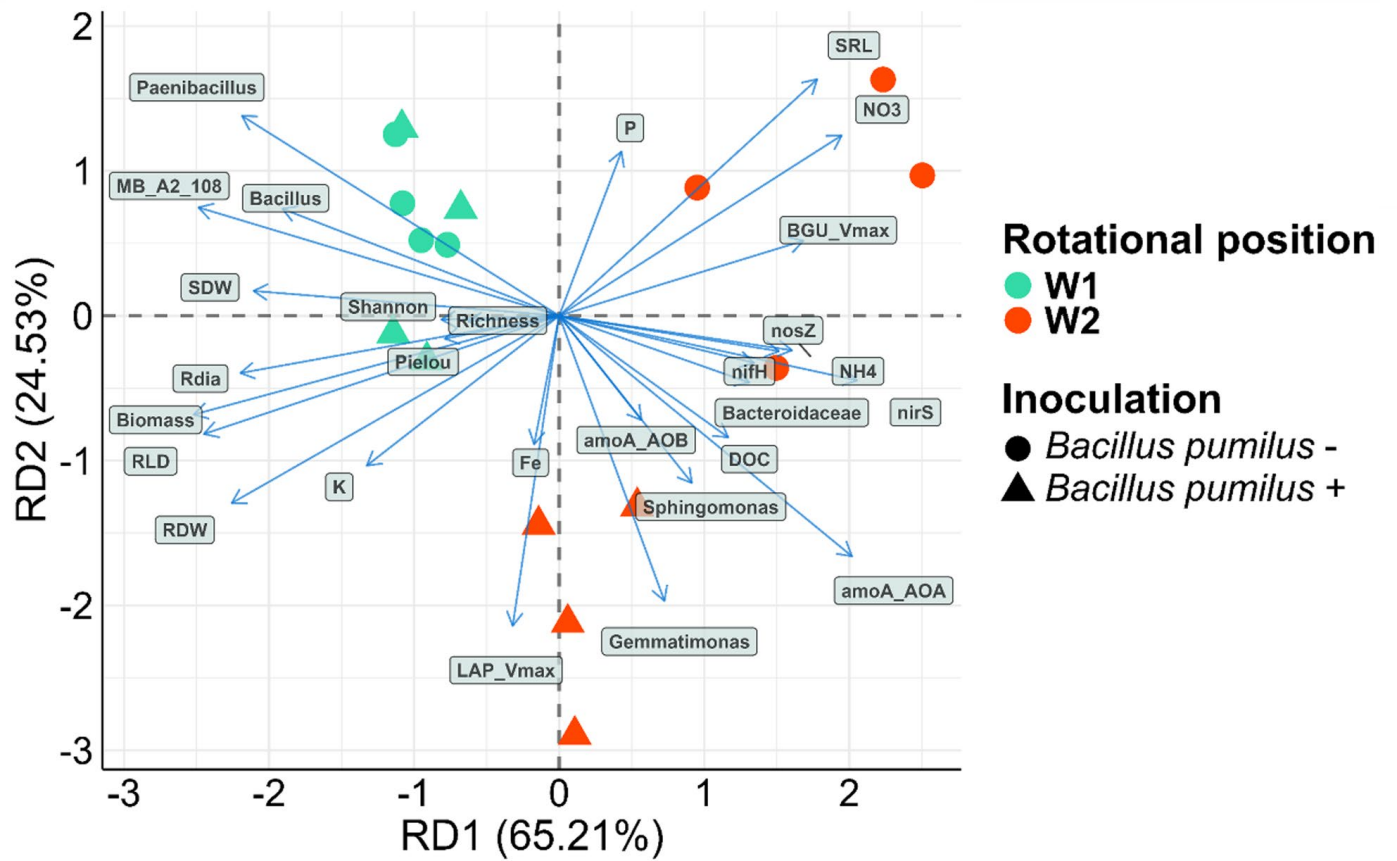
Redundancy analysis of the relationship between winter wheat total biomass, nutrient uptake, root growth, soil chemical properties and influential taxa in two rotational positions of winter wheat and two PGPR treatments at the end of tillering (BBCH 29). Colored circles represent the rotational positions of WW and the shape of the circles represents the PGPR inoculation treatment. BGU: *β-*glucosidase, LAP: leucine aminopeptidase, V_max_: maximum reaction rate, P: plant phosphorus, K: plant potassium, Fe: plant iron, R_dia_: average root diameter, RDW: root dry weight, RLD: root length density, SRL: specific root length, SDW: shoot dry weight, DOC: Dissolved organic carbon. Factors are represented by arrows; the length of the arrows indicates the influence that each factor exerts on the variation of the dataset

RD1 and RD2 explained 65.21% and 24.54% of the variation in the dataset across all soil depths, constrained by the rotational position of WW and *B. pumilus* seed coating (Fig. 5). SRL, soil NO^−^ _3_-N and BGU V_max_ were negatively associated with RLD, R_dia_, plant K% and total plant biomass, while *Paenibacillus*, MB-A2-108 and *Bacillus* were negatively associated with N-cycling genes, DOC, *Bacteroidaceae* and soil NH_4_^+^. Across both PGPR treatments, W1 and W2 plants were located on the opposite sides of RD1 axis, with the exception of one replicate of W2+. In the same axis, *B. pumilus* seed coating in W1 did not result in a separate clustering of the plants, with both W1- and W1 + clustering together on the negative portion of RD1. Contrarily, W2- clustered on the upper right portion of RD1, with the exception of one replicate. *B. pumilus* seed coating in W2 clustered the plants separately than W2- towards the negative portions of RD2. W1- and W1 + were more associated with the taxa *Paenibacillus*, MB-A2-108 and *Bacillus* as well as total plant biomass (especially shoot dry weight) and root growth traits (especially R_dia_, and RLD). To a lesser extent, two replicates of W1 + plants clustered closer to the alpha diversity indices. Three replicates of W2- plants grouped closer to SRL, soil NO^−^ _3_-N and BGU V_max_ while one replicate was more associated with soil NH_4_^+^, *Bacteroidaceae* and the N-cycling genes. W2 + plants clustered closer to LAP V_max_, the taxa *Gemmatimonas* and *Sphingomonas* and to a lesser extent, plant Fe% and *amoA* AOB.

## Discussion

### Root adaptations and plant performance due to the rotational position of WW and PGPR

Successively grown WW benefited from PGPR inoculation (W2+) and outgrew its non-inoculated counterpart (W2-). Changes in root morphology are crucial in understanding the total biomass differences of the rotational positions. Root plasticity is a major response that determines a plant’s overall fitness [50]. *B. pumilus* seed coating in W2 + improved the root growth, increased the proportion of larger R_dia_ classes and reduced the SRL. W1 + plants responded similarly to W1- and W2 + plants, with no further improvement of plant performance in the non-successive WW rotation. In a recent meta-analysis, it was shown that R_dia_ is negatively correlated with SRL and positively associated with median root lifespan, which indicates that roots with a higher R_dia_ invest heavily in belowground C allocation per unit root length to achieve sufficient nutrient and water uptake [51]. It has been shown that when WW is grown after oilseed rape, there is a higher and sustained belowground allocation of freshly assimilated C that is closely matched by root adaptations [23]. This sustained higher C allocation in non-successive WW rotations could be either a direct result of increased root biomass similar to what we observed here, but it could also be partly due to enhanced root exudation and root lifespan [23, 51]. Even though we did not quantify the abundance of the soil-borne fungus *Gaeumannomyces tritic*i in this study, enhanced root senescence due to root rot caused by this fungus could be a prominent mechanism leading to yield decline in successive WW rotations [16]. However, we did observe that the shift in the microbiome sustaining successively grown WW is less sufficient than WW after oilseed rape, which confirms previous findings reported by Braun-Kiewnick et al. [26]. There was a higher relative abundance of Saccharimonadales in W1- compared with W2-, which is negatively associated with *Tilletia* incidence in wheat and is stimulated by root exudation in maize [52, 53]. The same was true for *Paenarthrobacter*, which promoted wheat growth by increasing zinc uptake [54]. *Achromobacter* has been shown to promote root growth and induce IAA production and nitrogenase activity in rice [55]. *Pseudomonas* is a known PGPR genus of wheat, capable of P-solubilization, IAA production and outcompeting important fungal pathogens [56, 57]. The same is true for *Lysobacter* which has been appraised for its disease suppressiveness against a wide range of pathogens, which has been associated with a shift of soil microbiome rather than direct antagonism [10, 58]. On the other hand, *Terrabacter*, which we found to be more enriched in W2- compared with W1-, has been found at a higher relative abundance in soil with high occurrence of *Gaeumannomyces tritic*i [59]. Interestingly, we found a higher relative abundance of *Curtobacterium*, *Acidovorax*, *Bacillus*, *Paenibacillus* in the subsoil of W2- compared with W1- which could reveal the potential of successive WW rotations to function as a source of antagonistic bacteria for important WW pathogens, as suggested in [26].

### The effect of root adaptations on soil nutrient supply and microbial composition in the rhizosphere of WW

Our study revealed a clear link between root growth traits, mineral N availability and total plant biomass, with root length density and root biomass being negatively associated with SRL and NO_3_^−^ as revealed by the clustering of W2- closer to these variables in the RDA. We could therefore accept our first hypothesis on the basis of these data. Plants tend to increase their SRL in response to abiotic stresses and N limitation [60, 61]. In our experiment, SRL was negatively correlated with RLD, R_dia_, and total plant biomass, revealing that root adaptations are pivotal in enhancing plant growth in plant succession. During the early growth stages of WW, Kaloterakis et al. [17] showed that microbial immobilization of available mineral N, as shown by higher soil mineral N and reduced plant N uptake, is an important mechanism that limits N uptake by successively grown WW, which is translated into reduced total plant biomass. Higher soil NO_3_^−^ and increased *nirS* gene abundance that were not linked to enhanced plant N uptake in W2 provide evidence for this microbial immobilization effect. Plants try to overcome this transient N limitation by increasing their root absorptive surface and, therefore, soil exploration [62]. As hypothesized, these root adaptations in W2- were associated with a significant C cost that did not compensate for the total plant biomass reduction. Perhaps it was more than merely the C cost that caused the observed effects: differences in the microbial community have been shown to strongly modulate the outcome of WW self-succession, with a potential microbial dysbiosis in the rhizosphere of the successive WW rotations [17].

We expected an improved nutrient supply in W2+, which was evident in this study. *B. pumilus* seed coating partly improved plant N% and K% in the successive wheat rotation. For N%, this was mainly due to the higher N% in the stems of the plants that resulted in a higher quality plant biomass of W2 + with a lower C:N ratio. Increased plant Fe%, albeit not significant, with similar P% were measured in W2 + plants compared with W2-. P% was associated closer with W2- compared with W2+. RDA revealed that *B. pumilus* seed coating improved plant K uptake, translating into higher above and belowground biomass. Our data on soil mineral N dynamics are in accordance with Kaloterakis et al. [17], who found a higher soil NH_4_^+^ (a) and soil NO_3_^−^ in successive WW rotations than in WW after oilseed rape. They attributed this to the reduced nutrient uptake capacity of the successive WW plants that formed a less extensive root system and the transient microbial immobilization of available minerals early in the growing season. In our study, *B. pumilus* seed coating to both rotations led to a significant decrease in soil NO_3_^−^ compared with their non-inoculated counterparts. Four potential mechanisms arise from this observation. *B. pumilus* seed coating could have induced important changes in the microbial community composition of the two rotational positions of WW (such as reduced abundance of ammonia-oxidizing bacteria and/or archaea), leading to nitrification inhibition [63]. However, this did not appear to be the case since PGPR-treated plants did not exhibit a lower *amoA* gene abundance of AOB and AOA, which was also confirmed by the differential analysis. In addition, it has been suggested that the exudation of certain compounds in the rhizosphere of cereals, termed biological nitrification inhibitors, can suppress the activity of nitrifiers, increasing soil NH_4_^+^ and potentially plant N uptake [63–65]. However, this was not supported by our data. We measured an increase in soil NH_4_^+^ in W2 compared with W1, which was not associated with the bacterial inoculation. This could be related to the residue quality of the preceding crops. It has been shown that oilseed rape’s residues are quickly mineralized in the soil due to its lower C:N ratio than WW straw, which was evident in our experiment from the larger rhizosphere extent for BGU release [66]. BGU activity reflects the degradation of cellulose and release of glucose in the soil, while LAP activity is involved in the hydrolysis of leucine from peptides and thus, plant and microbial acquisition of N. Another mechanism suggested by the RDA results could be that the soil NO_3_^−^ was quickly depleted due to root growth promotion by *B. pumilus* that resulted in enhanced mineral N uptake by the plants [67]. Finally, we observed an inoculation-dependent shift in certain bacterial taxa in W2 + compared with W2-. A higher abundance of Rhodanobacteraceae, Moraxellaceae and *Pseudomonas* was found in W2 + compared with W2-, suggesting that *B*. *pumilus* seed coating influenced the selection of PGPR in the rhizosphere of successive WW rotations. RDA revealed that *Gemmatimonas* was strongly associated with W2- plants and it has been previously suggested to occur more frequently in healthy versus diseased soils, while at the same time improving nutrient cycling and uptake by the plants [68, 69]. Both *Gemmatimonas* and LAP_Vmax were strongly linked to W2+, suggesting that *B*. *pumilus* seed coating has the potential to change the structure of the rhizobiome and soil activity to the benefit of successively-grown WW. Our study showed that this effect is driven by factors different from the ones that underlie the W1- versus W2- relationship, i.e., the combination of root growth responses and the presence of Bacillus and *Paenibacillus*, highlighting the potential of *B*. *pumilus* as a PGPR for successive WW rotations. All four mechanisms can co-exist and exert some influence on the performance of WW. From an environmental perspective, the significant reduction of soil NO_3_^−^ could limit NO_3_^−^ leaching and groundwater pollution, crediting additional value to the *B. pumilus* seed coating in successive WW rotations.

### Soil enzymatic activity and its link to microbial community and N-cycling

We expected a more efficient N supply and plant uptake in W2 + compared with W2-, reflected in the higher abundance of N-cycling bacteria and/or archaea and nitrifying genes. The higher LAP activity in W2 + mitigated the soil N limitation during the early growth of the plants by mineralizing organic N. In turn, the plants responded by promoting root growth and nutrient acquisition, improving N uptake and total plant biomass accumulation. The overall effect of WW rotation and PGPR treatment on the nitrifying and denitrifying genes that were measured was insignificant across all depths. The higher *amoA* of AOA at greater depth and to a lesser extent *amoA* of AOB might have promoted the production of NO_3_^−^ in W2-, which was taken up by the plants at lower amounts than W1-, W1 + and W2+. We observed similar trends as Kaloterakis et al. [24] for N-cycling genes. *nirS* genes were significantly reduced in W1- compared with W2-, while no effect was evident for *nosZ* gene abundance. *nirS* genes are linked to the reduction of NO_2_^−^ to NO, which is then reduced to N_2_O during denitrification [70] and our data suggest a more active denitrification in W2- compared with W1- which was partly offset by *B. pumilus* seed coating. Interestingly, MB-A2-108 has a higher relative abundance in the soil of successive WW in Giongo et al. [22].

In the absence of strong responses in N-cycling genes, we investigated differences in soil microbial community and activity and found that *B. pumilus* seed coating differentially affects W2 + than W2-, which might relate to the growth promotion effect. Braun-Kiewnick et al. [26] showed that *B. pumilus* possesses a high extracellular hydrolytic enzyme and siderophore production capacity and is capable of increasing the production of proteases, glucanases, and cellulases in the soil. There are also reports of enhanced phosphatase and urease activity following *B. pumilus* inoculation in salt-tolerant rice [71]. Using zymography, we localized enzymatic hotspots and the rhizosphere extent for both BGU and LAP in the rhizosphere of the plants [72]. Kaloterakis et al. [17] found an increased BGU and LAP activity in WW grown after oilseed rape compared with successive WW rotations, which was not the case in this experiment, where there were no significant differences between W1- and W2-. Nevertheless, using zymography, we measured a higher rhizosphere extent for BGU on W1- than W2-, but not when the plants were inoculated. This suggests that W1- had a more extensive BGU activity in its rhizosphere, enhancing the breakdown of cellulose and other β-glucosides. PGPR-treated W2 + exhibited an increasing trend for LAP rhizosphere extent compared with W2- which was also significantly higher than W1- and W1+. LAP V_max_ was also a highly influential factor in our RDA, with W2 + clustering very close to it alongside *Gemmatimonas* and Fe%. PGPR inoculation in W2 + stimulated root growth and enriched the rhizosphere of the plants with microbial taxa that were more efficient in N mineralization and organic N turnover. In addition to the positive effect of *B. pumilus* seed coating on WW, distinct microbial community changes emerged following inoculation, providing insights into the ecological niche and functional role of this strain under contrasting WW rotations.

## Conclusion

In this study, we tested the hypothesis of whether the seed coating of WW with *B. pumilus* can compensate for the reduced performance of successively grown WW. Our hypothesis was confirmed, and the total plant biomass growth reduction in the successive WW rotation was significantly alleviated by *B. pumilus* seed coating. We further expected a more efficient N supply and plant uptake in PGPR-treated W2, which was not supported by our data. W2 + exhibited a high LAP activity, root growth, and K uptake, which translated into higher above- and belowground biomass. The microbial community of W2 + was selectively enriched in specific taxa with potentially beneficial properties. We demonstrated that the PGPR application is a sustainable practice that can improve WW productivity during early growth. Our results enhance our understanding of the potential of PGPR to modulate the direction of plant-soil interactions in successive WW rotations and compensate for the prevalent negative plant-soil feedback under such rotations.

## Supplementary Information

Supplementary Material 1.

## Data Availability

Raw sequencing data were deposited in NCBI’s Sequence Read Archive under BioProject PRJNA1238563. All data will be uploaded to the BonaRes Repository for Soil and Agricultural Research Data.

## Abbreviations

AOA: Ammonia-Oxidizing Archaea
AOB: Ammonia-Oxidizing Bacteria
AMO: Ammonia Monooxygenase
amoA: ammonia monooxygenase alpha subunit
BBCH: Biologische Bundesanstalt, Bundessortenamt und CHemische Industrie
BGU: β-Glucosidase
C: Carbon
CFU: Colony Forming Unit
C: N: Carbon to Nitrogen Ratio
DOC: Dissolved Organic Carbon
Fe: Iron
IAA: Indole-3-Acetic Acid
K: Potassium
K_m_: Michaelis Constant
LAP: Leucine Aminopeptidase
LMM: Linear Mixed-Effects Model
N: Nitrogen
NH_4_^+^: Ammonium
nirS: Nitrite Reductase
nifH: Nitrogenase
NO_3_^−^: Nitrate
nosZ: Nitrous Oxide Reductase
P: Phosphorus
PGPR: Plant Growth-Promoting Rhizobacteria
PSF: Plant-Soil Feedback
R_dia_: Root Diameter
RH: Rhizosphere soil
RLD: Root Length Density
SDW: Shoot Dry Weight
SO_4_^2−^: Sulfate
SRL: Specific Root Length
TN: Total Nitrogen
TOC: Total Organic Carbon
V_max_: Maximum Reaction Rate
W1: First Winter Wheat after oilseed rape
W1+: First Winter Wheat after oilseed rape, inoculated with Bacillus pumilus
W1-: First Winter Wheat after oilseed rape, non-inoculated
W2: Second Winter Wheat after oilseed rape
W2+: Second Winter Wheat after oilseed rape, inoculated with Bacillus pumilus
W2-: Second Winter Wheat after oilseed rape, non-inoculated
WW: Winter Wheat
