# Supplementary material for: Bacillus seed coating mitigates early growth reduction in successive winter wheat without altering rhizosphere bacterial and archaeal communities
Source: BMC Plant Biol. 2026 Jan 14;26:163. doi: 10.1186/s12870-026-08128-2 (PMC12849470; doi:10.1186/s12870-026-08128-2)
Supplement: Supplementary file 1 — Supplementary Material 1. [file 12870_2026_8128_MOESM1_ESM.docx]

Supplementary Information for

***Bacillus* seed coating mitigates early growth reduction in continuous winter wheat without altering rhizosphere bacterial and archaeal communities**

Nikolaos Kaloterakis^a^, Andrea Braun-Kiewnick^c^, Mehdi Rashtbari^b^, Adriana Giongo^c^, Doreen Babin^c^, Priscilla M. Zamberlan^c^, Bahar S. Razavi^b^, Kornelia Smalla^c^, Rüdiger Reichel^a^, Nicolas Brüggemann^a^

^a^ Institute of Bio- and Geosciences, Agrosphere (IBG-3), Forschungszentrum Jülich GmbH, 52428 Jülich, Germany

^b^ Department of Soil and Plant Microbiome, Institute for Phytopathology, Christian-Albrechts-University of Kiel, 24118, Kiel, Germany

^c^ Institute for Epidemiology and Pathogen Diagnostics, Julius Kühn Institute (JKI) – Federal Research Centre for Cultivated Plants, 38104, Braunschweig, Germany

* corresponding author: n.kaloterakis@fz-juelich.de; nikoskaloter@gmail.com

**
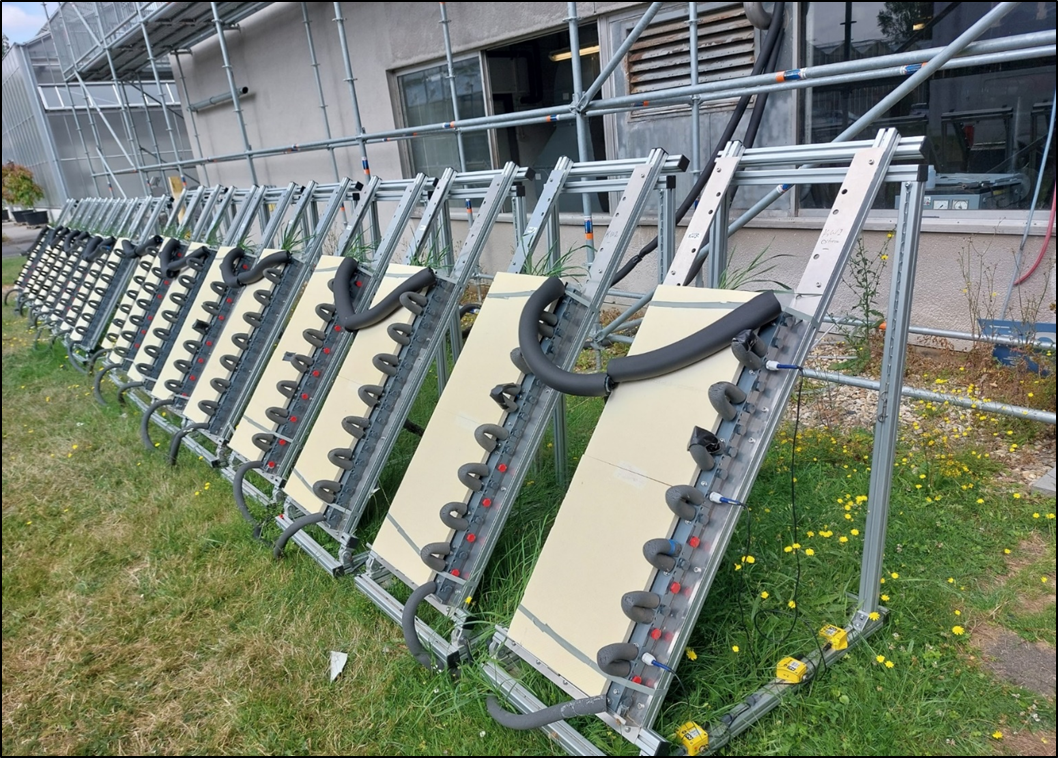
**

**Fig. A1**. Outdoor rhizotron experiment. The rhizotrons were connected to a cooling unit enabling the temperature regulation of each rhizotron.


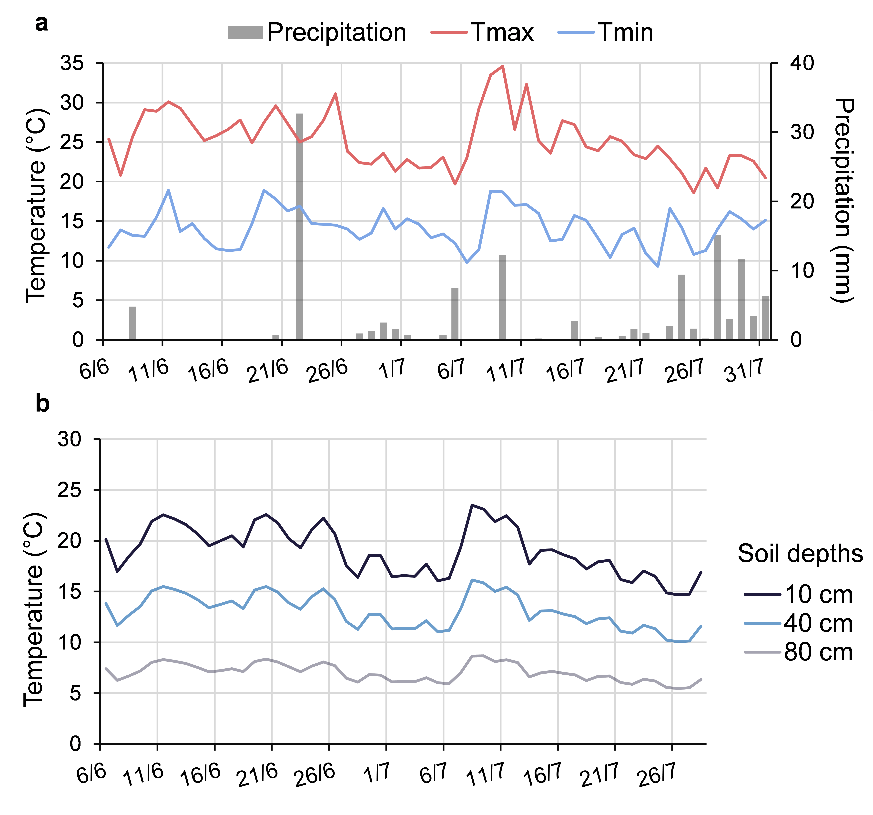


**Fig. A2**. Ambient minimum and maximum temperature and precipitation (a) during the experiment and soil temperature of the rhizotron at 10 cm, 40 cm, and 80 cm (b) during the experiment.


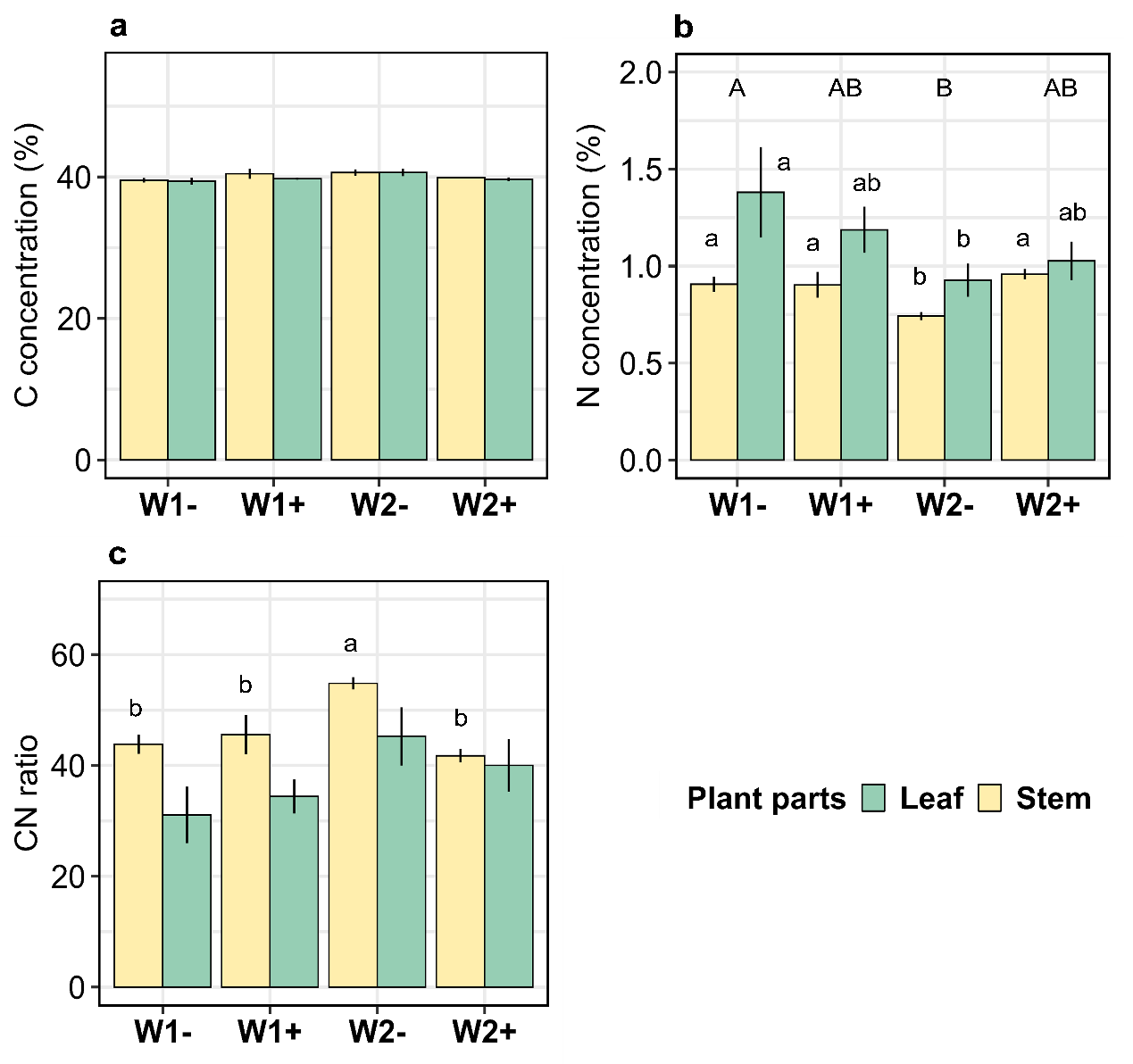


**Fig. A3**. Plant C concentration (a), N concentration (b), and C:N ratio (c) of leaves and stems of winter wheat at the end of tillering (BBCH 29). First wheat after oilseed rape without (W1-) and with (W1+) *Bacillus* *pumilus* and second wheat after oilseed rape without (W2-) and with (W2+) *Bacillus* *pumilus*. Different uppercase letters indicate significant differences between the rotational positions and PGPR treatments across all plant parts. For N concentration we used GLMM with gamma distribution. For C concentration and plant C:N ratio we used LMM using log-transformed data. Within each plant part, different lowercase letters indicate significant differences between the rotational positions and PGPR treatments at *p* ≤ 0.05 using the Benjamini-Hochberg adjustment for multiple comparisons. The absence of letters indicates non-significant differences.


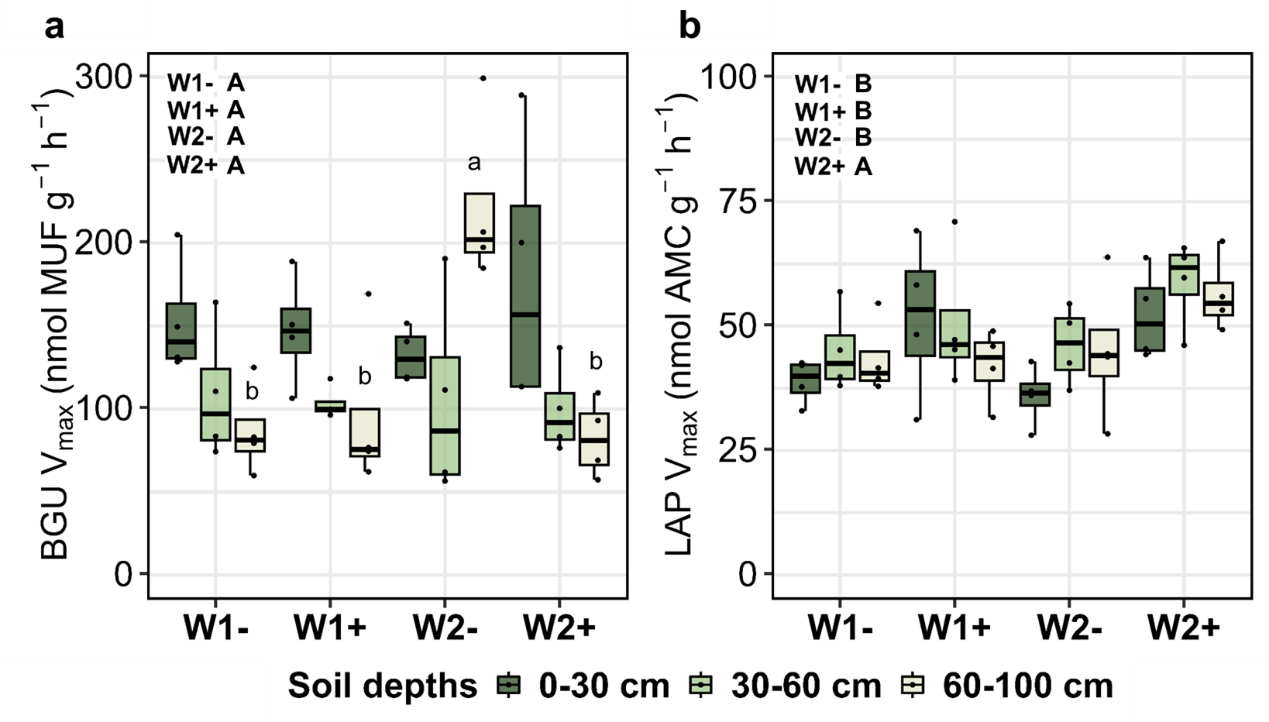


**Fig. A4**. Maximum reaction rate (V_max_) of *β*-glucosidase (BGU; a) and leucine aminopeptidase (LAP; b) of two rotational positions of winter wheat at the end of tillering (BBCH 29) at soil depths 0–30 cm, 30–60 cm, and 60–100 cm. First wheat after oilseed rape without (W1-) and with (W1+) *Bacillus* *pumilus* and second wheat after oilseed rape without (W2-) and with (W2+) *Bacillus* *pumilus*. Different uppercase letters indicate significant differences between the rotational positions and PGPR treatments across all soil depths. BGU V_max_ and LAP V_max_ were analyzed using LMM with log-normal distribution. Within each soil depth, different lowercase letters indicate significant differences between the rotational positions and PGPR treatments at *p* ≤ 0.05 using the Benjamini-Hochberg adjustment for multiple comparisons. The absence of letters indicates non-significant differences.


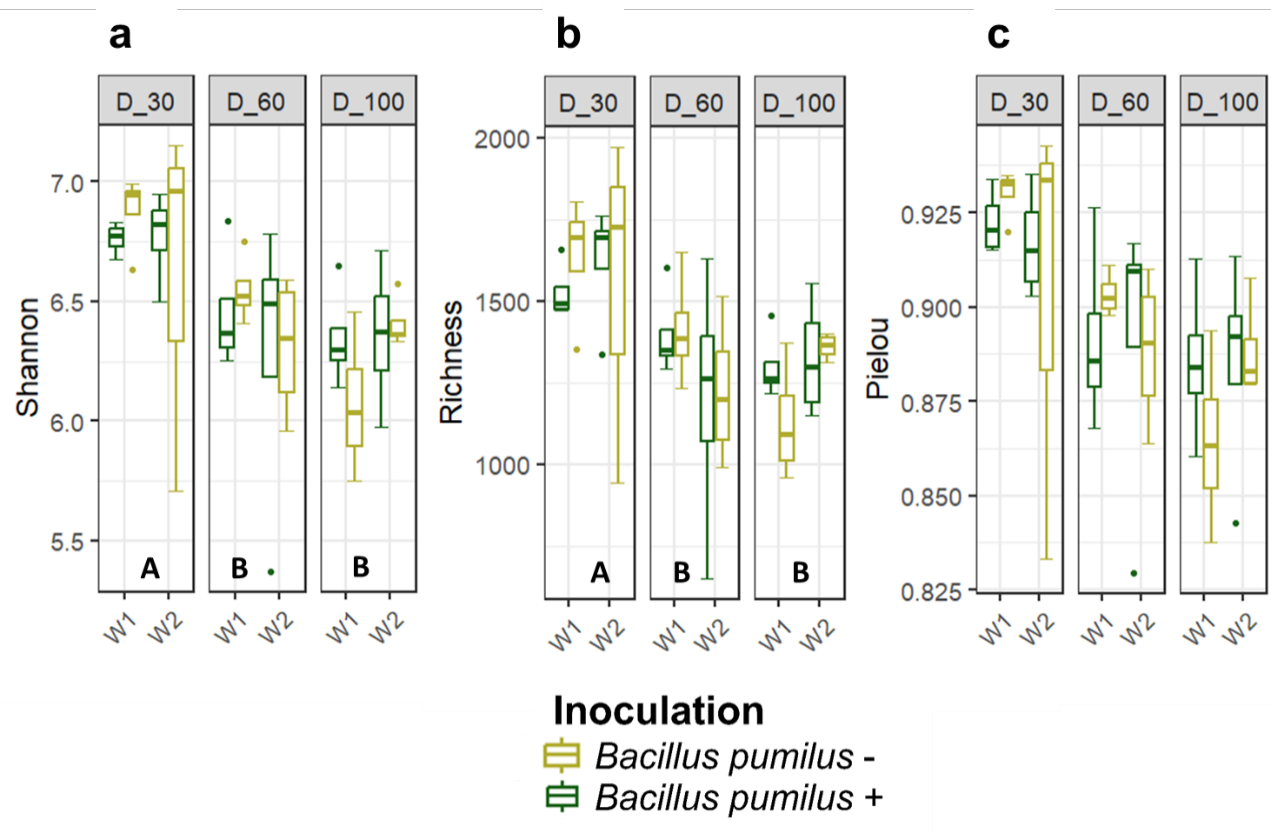


**Fig. A5**. Effect of the rotational position and PGPR inoculation on the microbial alpha diversity index Shannon (a) Richness (b) and Pielou of the following winter wheat at the end of tillering (BBCH 29), at soil depths 0–30 cm, 30–60 cm and 60–100 cm. First wheat after oilseed rape without (W1-) and with (W1+) *Bacillus* *pumilus* and second wheat after oilseed rape without (W2-) and with (W2+) *Bacillus* *pumilus*. Different uppercase letters denote significant differences between soil depths at p ≤ 0.05 according to the Kruskal Wallis test.


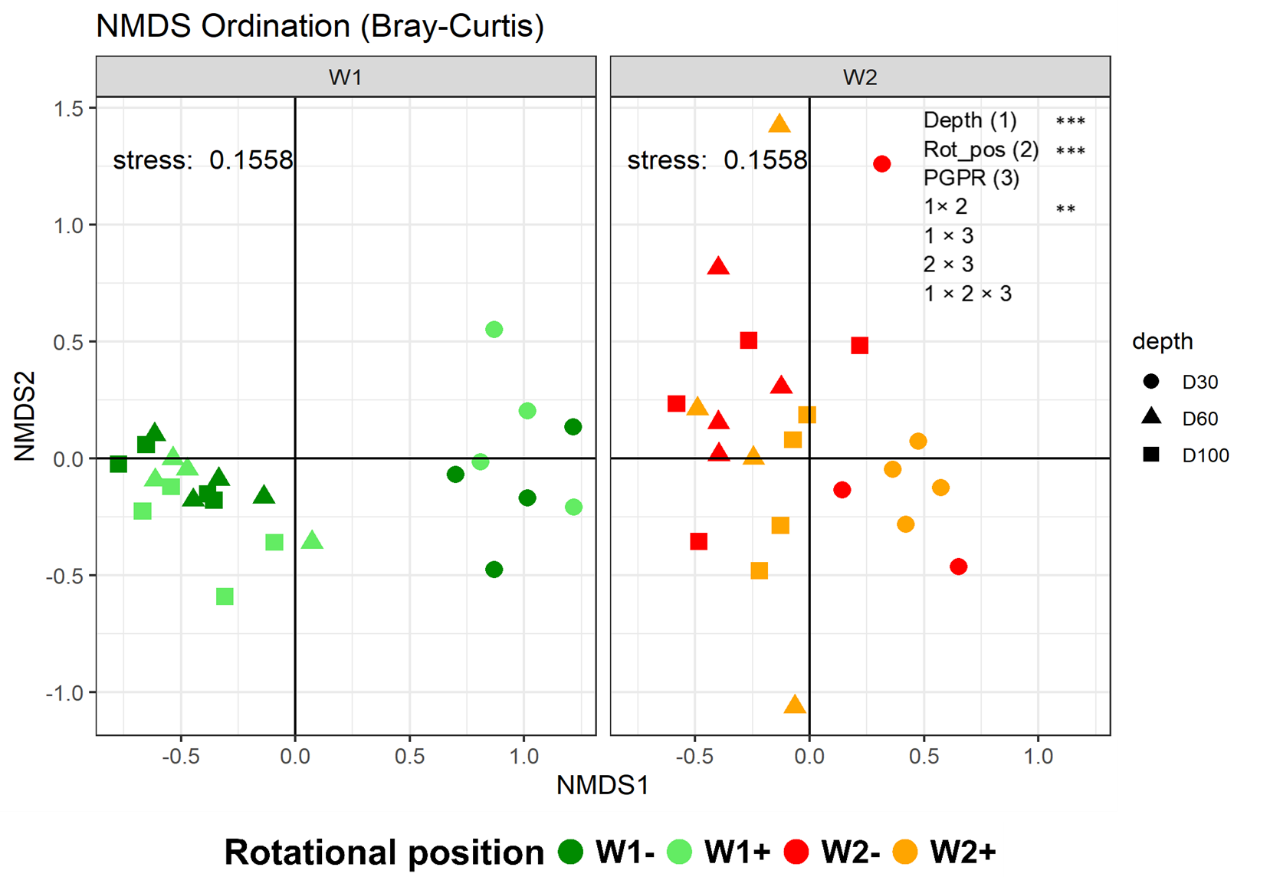


**Fig. A6**. Effect of the rotational position and PGPR inoculation on the beta diversity of the following winter wheat at the end of tillering (BBCH 29), at soil depths 0–30 cm, 30–60 cm and 60–100 cm. First wheat after oilseed rape without (W1-) and with (W1+) *Bacillus* *pumilus* and second wheat after oilseed rape without (W2-) and with (W2+) *Bacillus* *pumilus*. Beta diversity was measured using Bray-Curtis dissimilarity and visualized through non-metric multidimensional scaling (NMDS), with statistical analysis performed using PERMANOVA. Significant codes: 0 ‘***’, 0.001 ‘**’, 0.01 ‘*’, 0.05 ‘.’.


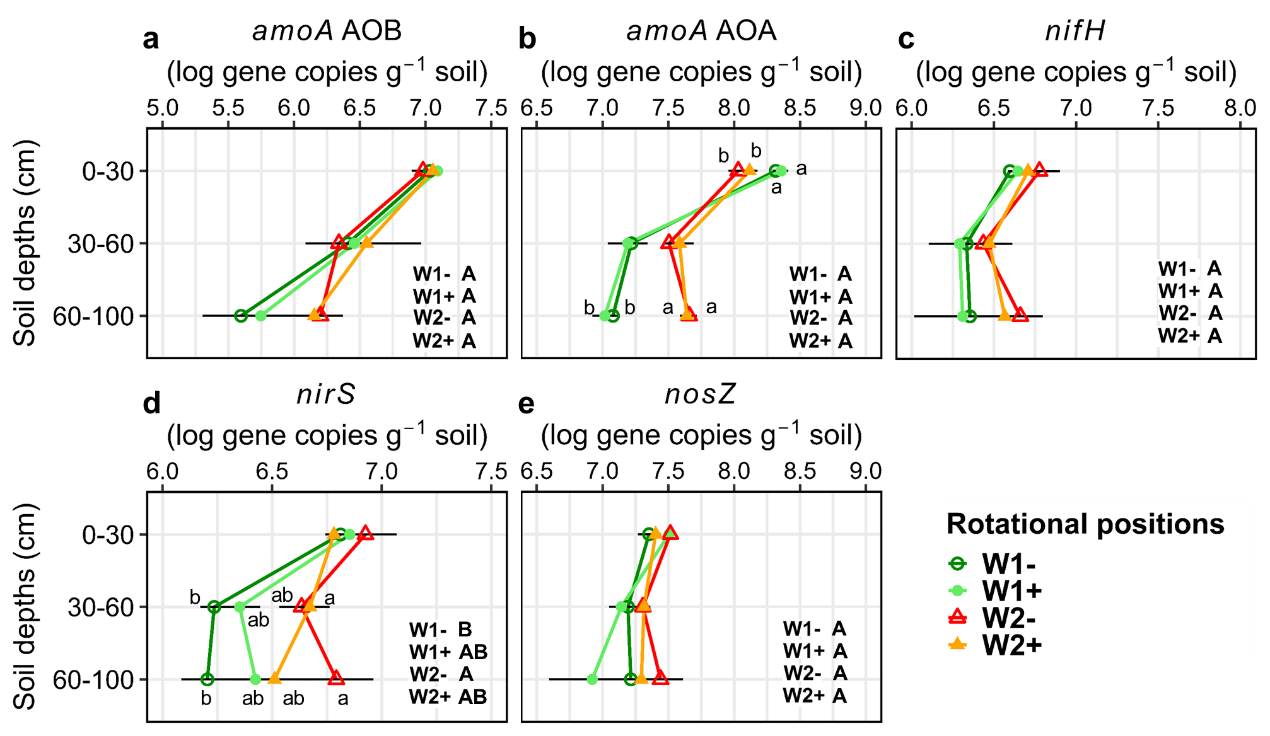


**Fig. A7**. Gene abundance of *amoA* AOB (a), *amoA* AOA (b), *nifH* (c), *nirS* (d) and *nosZ* (e) of two rotational positions of winter wheat at the end of tillering (BBCH 29) at soil depths 0–30 cm, 30–60 cm, and 60–100 cm. First wheat after oilseed rape without (W1-) and with (W1+) *Bacillus* *pumilus* and second wheat after oilseed rape without (W2-) and with (W2+) *Bacillus* *pumilus*. Different uppercase letters indicate significant differences between the rotational positions and PGPR treatments across all soil depths. *amoA* AOB, *amoA* AOA and *nirS* were analyzed using LMM. For *nifH* and *nosZ*, we used LMM with log-normal distribution. Within each soil depth, different lowercase letters indicate significant differences between the rotational positions and PGPR treatments at *p* ≤ 0.05 using the Benjamini-Hochberg adjustment for multiple comparisons. The absence of letters indicates non-significant differences.

**Table A1.** Effect of rotational position (Rot_pos), plant growth-promoting rhizobacterium (PGPR), plant part (Plant_part) and their interaction on plant dry weight (Biomass), plant carbon concentration (Plant C%), plant nitrogen concentration (Plant N%), plant carbon:nitrogen ratio (Plant CN), plant phosphorus concentration (Plant P%), plant potassium concentration (Plant K%) and plant iron concentration (Plant Fe%). Significant values at *p* ≤ 0.05 level are indicated in bold.

| ANOVA results |  | Biomass |  | Plant C% |  | Plant N% |  | Plant CN |  |
| --- | --- | --- | --- | --- | --- | --- | --- | --- | --- |
|  | df | Chisq | *p* value | Chisq | *p* value | Chisq | *p* value | Chisq | *p* value |
| Rot_pos | 1 | 0.281 | 0.596 | 0.021 | 0.885 | 1.664 | 0.197 | 1.207 | 0.272 |
| PGPR | 1 | 0.364 | 0.547 | 0.345 | 0.557 | 1.801 | 0.180 | 0.998 | 0.318 |
| Plant_part | 2 | 9.159 | **0.010** | 1.201 | 0.273 | 5.894 | **0.015** | 4.705 | **0.030** |
| Rot_pos × PGPR | 1 | 5.114 | **0.024** | 2.319 | 0.128 | 2.522 | 0.112 | 1.899 | 0.168 |
| Rot_pos × Plant_part | 2 | 5.947 | **0.050** | 0.233 | 0.629 | 1.656 | 0.198 | 1.462 | 0.227 |
| PGPR × Plant_part | 2 | 0.332 | 0.847 | 0.392 | 0.531 | 0.857 | 0.355 | 0.282 | 0.595 |
| Rot_pos × PGPR × Plant_part | 2 | 8.890 | **0.012** | 0.052 | 0.819 | 0.001 | 0.975 | 0.037 | 0.848 |
|  |  | Plant P% |  | Plant K% |  | Plant Fe% | |  |  |
|  | df | Chisq | *p* value | Chisq | *p* value | Chisq | *p* value |  |  |
| Rot_pos | 1 | 1.026 | 0.311 | 8.517 | **0.004** | 0.440 | 0.507 |  |  |
| PGPR | 1 | 3.354 | 0.067 | 18.067 | **0.000** | 1.281 | 0.258 |  |  |
| Plant_part | 2 | 3.267 | 0.071 | 42.387 | **0.000** | 0.001 | 0.971 |  |  |
| Rot_pos × PGPR | 1 | 0.035 | 0.851 | 34.687 | **0.000** | 0.160 | 0.689 |  |  |
| Rot_pos × Plant_part | 2 | 0.194 | 0.659 | 0.215 | 0.643 | 2.495 | 0.114 |  |  |
| PGPR × Plant_part | 2 | 4.977 | **0.026** | 3.793 | 0.051 | 0.636 | 0.425 |  |  |
| Rot_pos × PGPR × Plant_part | 2 | 2.364 | 0.124 | 6.105 | **0.013** | 0.175 | 0.676 |  |  |

**Table A2.** Effect of rotational position (Rot_pos), plant growth-promoting rhizobacterium (PGPR), soil depth (Depth) and their interaction on root dry weight (RDW), root length density (RLD), average root diameter (R_dia_), specific root length (SRL). Significant values at *p* ≤ 0.05 level are indicated in bold.

| ANOVA results |  | RDW |  | RLD |  | R_dia_ |  | SRL |  |
| --- | --- | --- | --- | --- | --- | --- | --- | --- | --- |
|  |  | Chisq | *p* value | Chisq | *p* value | Chisq | *p* value | Chisq | *p* value |
| Rot_pos | 1 | 2.858 | **0.091** | 1.358 | 0.244 | 12.974 | **0.000** | 5.070 | **0.024** |
| PGPR | 1 | 1.891 | 0.169 | 0.006 | 0.939 | 0.186 | 0.666 | 1.822 | 0.177 |
| Depth | 2 | 11.485 | **0.003** | 16.849 | **0.000** | 14.955 | **0.001** | 12.655 | **0.002** |
| Rot_pos × PGPR | 1 | 27.652 | **0.000** | 16.425 | **0.000** | 8.932 | **0.003** | 2.672 | 0.102 |
| Rot_pos × Depth | 2 | 0.847 | 0.655 | 0.845 | 0.656 | 12.656 | **0.002** | 2.444 | 0.295 |
| PGPR × Depth | 2 | 0.596 | 0.742 | 1.744 | 0.418 | 0.405 | 0.817 | 0.589 | 0.745 |
| Rot_pos × PGPR × Depth | 2 | 0.809 | 0.667 | 6.829 | **0.033** | 25.201 | **0.000** | 3.579 | 0.167 |

**Table A3.** Effect of rotational position (Rot_pos), plant growth-promoting rhizobacterium (PGPR), soil depth (Depth) and their interaction on the proportion of total root length in the < 0.1 mm diameter class (P1), in the 0.1–0.2 mm diameter class (P2), in the 0.2–0.3 mm diameter class (P3), in the 0.3–0.4 mm diameter class (P4), in the 0.4–0.5 mm diameter class (P5), in the > 0.5 mm diameter class (P6). Significant values at *p* ≤ 0.05 level are indicated in bold.

|  |  | P1 |  | P2 |  | P3 |  |
| --- | --- | --- | --- | --- | --- | --- | --- |
|  |  | Chisq | *p* value | Chisq | *p* value | Chisq | *p* value |
| Rot_pos | 1 | 13.304 | **0.000** | 0.132 | 0.716 | 17.715 | **0.000** |
| PGPR | 1 | 0.131 | 0.718 | 0.004 | 0.951 | 0.131 | 0.717 |
| Depth | 2 | 7.142 | **0.028** | 45.449 | **0.000** | 6.823 | **0.033** |
| Rot_pos × PGPR | 1 | 1.430 | 0.232 | 0.597 | 0.440 | 0.677 | 0.410 |
| Rot_pos × Depth | 2 | 13.376 | 0.001 | 24.352 | **0.000** | 9.477 | **0.009** |
| PGPR × Depth | 2 | 0.088 | 0.957 | 3.358 | 0.187 | 1.292 | 0.524 |
| Rot_pos × PGPR × Depth | 2 | 11.382 | **0.003** | 9.543 | **0.008** | 1.671 | 0.434 |
|  |  | P4 |  | P5 |  | P6 |  |
|  |  | Chisq | *p* value | Chisq | *p* value | Chisq | *p* value |
| Rot_pos | 1 | 21.498 | **0.000** | 18.195 | **0.000** | 22.698 | **0.000** |
| PGPR | 1 | 0.096 | 0.757 | 0.061 | 0.805 | 0.193 | 0.661 |
| Depth | 2 | 11.886 | **0.003** | 28.722 | **0.000** | 27.513 | **0.000** |
| Rot_pos × PGPR | 1 | 0.099 | 0.753 | 1.767 | 0.184 | 34.211 | 0.000 |
| Rot_pos × Depth | 2 | 16.613 | **0.000** | 10.254 | **0.006** | 25.743 | **0.000** |
| PGPR × Depth | 2 | 0.231 | 0.891 | 2.044 | 0.360 | 2.233 | 0.327 |
| Rot_pos × PGPR × Depth | 2 | 4.932 | 0.085 | 7.879 | **0.019** | 63.117 | **0.000** |

**Table A4.** Effect of rotational position (Rot_pos), plant growth-promoting rhizobacterium (PGPR), soil depth (Depth) and their interaction on ammonium (N-NH_4_^+^), nitrate (N-NO_3_^-^) and dissolved organic C (DOC). Significant values at *p* ≤ 0.05 level are indicated in bold.

| ANOVA results |  | N-NH_4_^+^ |  | N-NO_3_^-^ |  | DOC |  |
| --- | --- | --- | --- | --- | --- | --- | --- |
|  | df | Chisq | *p* value | Chisq | *p* value | Chisq | p value |
| Rot_pos | 1 | 5.688 | **0.017** | 0.438 | 0.508 | 0.585 | 0.444 |
| PGPR | 1 | 1.267 | 0.260 | 1.323 | 0.250 | 0.190 | 0.663 |
| Depth | 2 | 1.530 | 0.465 | 26.117 | **0.000** | 0.788 | 0.674 |
| Rot_pos × PGPR | 1 | 0.058 | 0.809 | 0.523 | 0.470 | 0.479 | 0.489 |
| Rot_pos × Depth | 2 | 0.099 | 0.952 | 11.307 | **0.004** | 1.592 | 0.451 |
| PGPR × Depth | 2 | 1.808 | 0.405 | 29.078 | **0.000** | 2.521 | 0.284 |
| Rot_pos × PGPR × Depth | 2 | 2.606 | 0.272 | 5.671 | 0.059 | 2.782 | 0.249 |

**Table A5.** Effect of rotational position (Rot_pos), plant growth-promoting rhizobacterium (PGPR), soil depth (Depth) and their interaction on *β*-glucosidase (BGU) hotspot percent, BGU activity, BGU RH extent, BGU maximum reaction rate (BGU V_max_), BGU substrate affinity (BGU K_m_), leucine aminopeptidase (LAP) hotspot percent, LAP activity, LAP RH extent, maximum reaction rate (LAP V_max_) and LAP substrate affinity (LAP K_m_). Significant values at *p* ≤ 0.05 level are indicated in bold.

| ANOVA results |  | BGU hotspot | | BGU RH extent | | BGU activity | | BGU V_max_ |  | BGU K_m_ |  |
| --- | --- | --- | --- | --- | --- | --- | --- | --- | --- | --- | --- |
|  | df | Chisq | *p* value | Chisq | *p* value | Chisq | *p* value | Chisq | *p* value | Chisq | *p* value |
| Rot_pos | 1 | 0.726 | 0.394 | 0.100 | 0.751 | 0.977 | 0.323 | 0.380 | 0.538 | 0.573 | 0.449 |
| PGPR | 1 | 0.073 | 0.787 | 10.281 | **0.001** | 0.019 | 0.890 | 0.040 | 0.842 | 1.058 | 0.304 |
| Depth | 2 | 3.349 | 0.187 | 0.060 | 0.970 | 1.528 | 0.466 | 5.296 | 0.071 | 1.069 | 0.586 |
| Rot_pos × PGPR | 1 | 0.141 | 0.707 | 6.659 | **0.010** | 0.417 | 0.518 | 0.763 | 0.382 | 9.571 | **0.002** |
| Rot_pos × Depth | 2 | 1.513 | 0.469 | 0.812 | 0.666 | 1.269 | 0.530 | 0.668 | 0.716 | 7.255 | **0.027** |
| PGPR × Depth | 2 | 2.647 | 0.266 | 0.708 | 0.702 | 3.086 | 0.214 | 0.091 | 0.955 | 2.231 | 0.328 |
| Rot_pos × PGPR × Depth | 2 | 2.573 | 0.276 | 0.750 | 0.687 | 0.332 | 0.847 | 10.462 | **0.005** | 18.797 | **0.000** |
|  |  | LAP hotspot | | LAP RH extent | | LAP activity | | LAP Vmax | | LAP Km |  |
|  |  | Chisq | *p* value | Chisq | *p* value | Chisq | *p* value | Chisq | *p* value | Chisq | *p* value |
| Rot_pos | 1 | 2.931 | 0.087 | 2.127 | 0.145 | 0.016 | 0.899 | 0.07 | 0.792 | 1.778 | 0.182 |
| PGPR | 1 | 1.608 | 0.205 | 0.029 | 0.865 | 0.659 | 0.417 | 2.79 | 0.095 | 0.036 | 0.849 |
| Depth | 2 | 7.781 | **0.020** | 4.332 | 0.115 | 9.652 | **0.008** | 1.87 | 0.392 | 3.192 | 0.203 |
| Rot_pos × PGPR | 1 | 1.246 | 0.264 | 0.107 | 0.744 | 0.120 | 0.729 | 0.33 | 0.563 | 0.390 | 0.532 |
| Rot_pos × Depth | 2 | 0.932 | 0.627 | 0.107 | 0.948 | 1.338 | 0.512 | 1.52 | 0.467 | 3.377 | 0.185 |
| PGPR × Depth | 2 | 3.302 | 0.192 | 1.501 | 0.472 | 1.573 | 0.455 | 1.81 | 0.405 | 0.656 | 0.720 |
| Rot_pos × PGPR × Depth | 2 | 0.633 | 0.729 | 2.624 | 0.269 | 1.883 | 0.390 | 0.39 | 0.823 | 2.495 | 0.287 |

**Table A6.** Effect of rotational position (Rot_pos), plant growth-promoting rhizobacterium (PGPR), soil depth (Depth), and their interaction on Shannon, Richness and Pielou indices. Significant values at *p* ≤ 0.05 level are indicated in bold.

| ANOVA results |  | Shannon |  | Richness |  | Pielou |  |
| --- | --- | --- | --- | --- | --- | --- | --- |
|  | df | Chisq | *p* value | Chisq | *p* value | Chisq | *p* value |
| Rot_pos | 1 | 0.246 | 0.620 | 0.018 | 0.893 | 4.062 | **0.044** |
| PGPR | 1 | 0.030 | 0.863 | 0.048 | 0.827 | 0.539 | 0.463 |
| Depth | 2 | 51.878 | **0.000** | 25.311 | **0.000** | 121.529 | **0.000** |
| Rot_pos × PGPR | 1 | 0.670 | 0.413 | 0.002 | 0.965 | 0.000 | 0.999 |
| Rot_pos × Depth | 2 | 4.363 | 0.113 | 1.444 | 0.486 | 16.644 | **0.000** |
| PGPR × Depth | 2 | 0.223 | 0.895 | 0.088 | 0.957 | 0.966 | 0.617 |
| Rot_pos × PGPR × Depth | 2 | 1.002 | 0.606 | 0.460 | 0.795 | 2.387 | 0.303 |

| ANOVA results |  | 16S rRNA Bacteria | | *amoA* AOB | | 16S rRNA Archaea | | *amoA* AOA | |
| --- | --- | --- | --- | --- | --- | --- | --- | --- | --- |
|  | df | Chisq | *p* value | Chisq | *p* value | Chisq | *p* value | Chisq | *p* value |
| Rot_pos | 1 | 0.246 | 0.620 | 0.018 | 0.893 | 4.062 | **0.044** | 5.271 | **0.022** |
| PGPR | 1 | 0.030 | 0.863 | 0.048 | 0.827 | 0.539 | 0.463 | 0.161 | 0.688 |
| Depth | 2 | 51.878 | **0.000** | 25.311 | **0.000** | 121.529 | **0.000** | 193.642 | **0.000** |
| Rot_pos × PGPR | 1 | 0.670 | 0.413 | 0.002 | 0.965 | 0.000 | 0.999 | 0.088 | 0.767 |
| Rot_pos × Depth | 2 | 4.363 | 0.113 | 1.444 | 0.486 | 16.644 | **0.000** | 36.430 | **0.000** |
| PGPR × Depth | 2 | 0.223 | 0.895 | 0.088 | 0.957 | 0.966 | 0.617 | 0.507 | 0.776 |
| Rot_pos × PGPR × Depth | 2 | 1.002 | 0.606 | 0.460 | 0.795 | 2.387 | 0.303 | 0.127 | 0.938 |
|  |  | *nifH* |  | *nirS* |  | *nosZ* |  |  |  |
|  |  | Chisq | *p* value | Chisq | *p* value | Chisq | *p* value |  |  |
| Rot_pos | 1 | 0.023 | 0.880 | 0.247 | 0.619 | 0.298 | 0.585 |  |  |
| PGPR | 1 | 0.050 | 0.822 | 0.082 | 0.774 | 0.680 | 0.410 |  |  |
| Depth | 2 | 3.980 | 0.137 | 14.072 | **0.001** | 11.537 | **0.003** |  |  |
| Rot_pos × PGPR | 1 | 0.198 | 0.656 | 0.820 | 0.365 | 1.012 | 0.314 |  |  |
| Rot_pos × Depth | 2 | 0.587 | 0.746 | 3.719 | 0.156 | 3.835 | 0.147 |  |  |
| PGPR × Depth | 2 | 0.166 | 0.920 | 0.763 | 0.683 | 3.486 | 0.175 |  |  |
| Rot_pos × PGPR × Depth | 2 | 0.277 | 0.871 | 2.313 | 0.315 | 1.494 | 0.474 |  |  |

**Table A7.** Effect of rotational position (Rot_pos), plant growth-promoting rhizobacterium (PGPR), soil depth (Depth), and their interaction on 16S rRNA of Bacteria, 16S rRNA of Archaea, *amoA* of ammonia oxidizing bacteria (*amoA* AOB) and archaea (*amoA* AOA), *nifH*, *nirS* and *nosZ* gene abundance. Significant values at *p* ≤ 0.05 level are indicated in bold.
